# Metabolomic Analysis of Histological Composition Variability of High-Grade Serous Ovarian Cancer Using ^1^H HR MAS NMR Spectroscopy

**DOI:** 10.3390/ijms252010903

**Published:** 2024-10-10

**Authors:** Agnieszka Skorupa, Mateusz Klimek, Mateusz Ciszek, Sławomir Pakuło, Tomasz Cichoń, Bartosz Cichoń, Łukasz Boguszewicz, Andrzej Witek, Maria Sokół

**Affiliations:** 1 Department of Medical Physics, Maria Skłodowska-Curie National Research Institute of Oncology, Gliwice Branch, 44-102 Gliwice, Poland; mateusz.ciszek@gliwice.nio.gov.pl (M.C.); lukasz.boguszewicz@gliwice.nio.gov.pl (Ł.B.); maria.sokol@gliwice.nio.gov.pl (M.S.); 2 Department of Gynecology, Obstetrics and Oncological Gynecology, Faculty of Medicine in Katowice, Medical University of Silesia, 40-752 Katowice, Poland; matheaus.klimek@gmail.com (M.K.); cichon.tomasz.ct@gmail.com (T.C.); bartosz.cichon23@gmail.com (B.C.); awitek@sum.edu.pl (A.W.); 3 Tumor Pathology Department, Maria Skłodowska-Curie National Research Institute of Oncology, Gliwice Branch, 44-102 Gliwice, Poland; slawomir.pakulo@gliwice.nio.gov.pl

**Keywords:** high-grade serous ovarian cancer, metabolomics, high-resolution magic-angle spinning nuclear magnetic resonance spectroscopy

## Abstract

In this work, the HR MAS NMR (high-resolution magic-angle spinning nuclear magnetic resonance) spectroscopy technique was combined with standard histological examinations to investigate the metabolic features of high-grade serous ovarian cancer (HGSOC) with a special focus on the relation between a metabolic profile and a cancer cell fraction. The studied group consisted of 44 patients with HGSOC and 18 patients with benign ovarian tumors. Normal ovarian tissue was also excised from 13 control patients. The metabolic profiles of 138 tissue specimens were acquired on a Bruker Avance III 400 MHz spectrometer. The NMR spectra of the HGSOC samples could be discriminated from those acquired from the non-transformed tissue and were shown to depend on tumor purity. The most important features that differentiate the samples with a high fraction of cancer cells from the samples containing mainly fibrotic stroma are the increased intensities in the spectral regions corresponding to phosphocholine/glycerophosphocholine, phosphoethanolamine/serine, threonine, uridine nucleotides and/or uridine diphosphate (UDP) nucleotide sugars. Higher levels of glutamine, glutamate, acetate, lysine, alanine, leucine and isoleucine were detected in the desmoplastic stroma within the HGSOC lesions compared to the stroma of benign tumors. The HR MAS NMR analysis of the metabolic composition of the epithelial and stromal compartments within HGSOC contributes to a better understanding of the disease’s biology.

## 1. Introduction

Globally, ovarian cancer (OC) is the third most frequently diagnosed gynecological cancer [1] and the eighth leading cause of death from any cancer in women [2]. The most common histological type is high-grade serous ovarian cancer (HGSOC) that appears to arise from the ovary, fallopian tube, or peritoneum [3]. This type is diagnosed in 75% of ovarian cancer cases. The silent evolution of the disease and the lack of effective screening tests for this cancer translate directly into a low early detection rate (below 25% of cases). Although most patients respond well to the standard treatment (cytoreductive surgery and chemotherapy), disease recurrence and acquired resistance to chemotherapeutics are very common and eventually lead to patient death. Despite the novel therapeutic options (the poly(ADP-ribose) polymerase (PARP) inhibitors [4] and antiangiogenic therapy [5]), the treatment results are not satisfactory [6]. A deeper understanding of the disease at the molecular level is necessary for the identification of potential therapeutic targets and the stratification of the patients.

The vast majority of proteomic and genomic studies of ovarian cancer performed to date exploited bulk tissue analysis techniques [7,8]. In particular, the Cancer Genome Atlas (TCGA) project demonstrated that HGSOC is molecularly heterogeneous and could be classified into four subtypes: mesenchymal, immunoreactive, proliferative, and differentiated [7]. The mesenchymal subtype (characterized by a high content of stromal cells) is associated with short overall survival, while the immunoreactive one (revealing upregulation of the genes involved in immune cell activation) featured a better outcome [9,10]. Although the prognostic relevance of these subtypes and the classifications developed by other groups is still debated [11], it is generally accepted that the tumor microenvironment, TME (which contributes significantly to these subtypes), plays an important role in the tumor initiation and progression [12]. In addition to complex molecular signatures, a simple histological parameter, such as the tumor to stroma ratio, was also shown to be of prognostic importance in ovarian cancer [13,14]. Schwede et al. claim that proper accounting for the nonmalignant component in the molecular analysis of bulk tissues is mandatory for correct interpretation of the results [15]. In their work, the assignment of ovarian tumors to the appropriate molecular subtypes was shown to be affected by the percentage content of stroma in the samples, while a proper adjustment for this factor revealed that a stromal gene expression was associated with overall survival.

Although it is well known that tumors are heterogeneous and bulk tissue analysis is prone to sample selection bias, Swift et al. reported that in 42% of the reviewed genetic/biomarker studies, no information on samples purity thresholds was provided, while a great variability of this parameter was observed in the remaining works (from 10 to 100%) [16]. It is now clear that molecular profiling in the search for diagnostic and prognostic biomarkers should be performed with the use of techniques that enable the correlation of the obtained results with a standard histology.

High-resolution magic-angle spinning nuclear magnetic resonance (HR MAS NMR) is a high-throughput method that allows the determination of the metabolic profile of intact tissue without the need for homogenization and extraction [17]. After NMR analysis, the sample can be subjected to pathological examination. This approach was successfully used to find the relationship between tumor purity and metabolic profile in breast tumors [18] and prostate tumors [19]. Although HR MAS NMR revealed its usefulness in metabolic studies of various tumors [20], the application of this method is reported in only two works devoted to ovarian cancer [21,22]. Ricci et al. focused on the influence of sample freezing time on HR MAS NMR profiles of ovarian tumors [21], while Ben Sellem et al. [22] showed the usefulness of the method in discriminating between different histological types of ovarian carcinoma and attempted to construct statistical models to predict survival rates and response to therapy in patients with serous cancer. However, in the latter work, the samples were identified as pathological on the basis of clinical diagnoses and not due to a direct histopathological analysis performed after the HR MAS NMR measurement.

The purpose of this work was to combine the metabolic profiles obtained with HR MAS NMR with standard histological examinations of the post-HR MAS NMR samples of HGSOC, benign ovarian tumors, and normal ovarian tissue to characterize the metabolic composition of the epithelial and stromal compartments in ovarian lesions. Focusing on the important role of the stromal compartment in ovarian cancer progression and metastasis, particular attention was paid to the comparison of the metabolic characteristics of the stroma within the cancerous and non-malignant lesions.

## 2. Results

### 2.1. Studied Group and Tissue Samples

A total of 44 patients diagnosed with high-grade serous ovarian cancer (HGSOC), 18 patients diagnosed with benign ovarian tumors, and 13 control patients, all hospitalized in the Department and Clinic of Gynecology and Obstetrics of the Medical University of Silesia in Katowice, were enrolled in the study. The clinical–pathological characteristics of the studied groups are presented in Table 1. A Kruskal–Wallis test revealed no differences in BMI and age between the analyzed classes (*p* > 0.05). In each group more than 60% of the patients were post-menopausal.

The HR MAS NMR spectra were acquired from the following patients:

- Single tissue samples were obtained from 21 patients with malignant tumors, 14 patients with benign tumors, and 13 control patients;
- Samples were excised bilaterally from 3 patients with malignant tumors and from 3 patients with benign tumors;
- Multiple samples were obtained from neighboring regions of malignant tumors in 20 patients and in 1 patient with a serous cystadenofibroma ovary (2–5 samples per patient).

The tumor samples collected from adjacent 4–5 locations constitute a ‘heterogeneity dataset’. This dataset was used to assess intra-tumor and inter-patient metabolic variabilities.

After the ^1^H HR-MAS NMR measurements, the samples were frozen for histopathological evaluation of the tissue content.

### 2.2. Post-^1^H HR-MAS NMR Histopathology

Table 2 and Figure S1 show the results obtained from the histological evaluation of the samples previously analyzed with HR MAS NMR. Cancer cells were not present in 17 specimens obtained from 11 patients with malignant tumors. These samples (C 0%) were found to contain mainly normal ovarian tissue (median 75%). A total of 22 samples (C 1–19%) from 20 patients were characterized by a cancer purity of 1 to 19% (median 5%). This group of the samples was also found to be contaminated with normal ovary tissue (median 50%). The percentage of cancer cells exceeded 20% for 55 samples from 23 patients (C 20–100%). The median cancer content in the latter group of the samples was 40%. These samples also contained a significant portion of the fibrotic stroma (median 45%). The specimens collected from the control group were dominated by a normal ovarian tissue component (median 100%).

Histopathological analysis revealed that 11 samples excised from 6 patients with benign serous masses and 1 fibroma contained mainly intra-tumoral fibrous tissue (median 90%). The epithelial tissue component was shown to have a negligible contribution to the overall tumor volume in these samples. Table 2 provides detailed information about the histological composition of these specimens. Furthermore, nine samples from eight patients diagnosed with various benign lesions were found to contain mainly non-tumoral fibrous connective tissue or fibrous tumor capsule (median fibrous connective tissue in these samples was 82.5%, not included in Table 2). Three samples collected from three patients with benign tumors (simple ovarian cyst, mucinous cystadenofibroma, and ovarian fibroma) were representative of corpus albicans (more than 60% of the total sample volume) and one sample from the patient with serous cystadenofibroma was representative of normal ovarian tissue (100% tumor volume). These samples are also not included in Table 2.

The data presented indicate that such components as inflammation, calcification, and vessels, as well as an epithelial component in benign tumors, are not well represented in the studied material (Table 2). However, several samples were shown to contain a significant fraction of necrosis.

To facilitate comparison of the metabolic properties of different tissue types, the analyzed samples were considered representative of cancer (HGSOC), fibrotic compartment within malignant tumors, fibrotic compartment within benign tumors, normal ovary tissue (the samples collected from the control group), normal ovary tissue (the samples collected from the cancer patients), necrosis, and non-tumoral fibrous tissue (obtained from the patients with benign non-neoplastic lesions) according to the criteria presented in Table 3. The exemplary images of the samples stained with hematoxylin and eosin are presented in Figure S2.

### 2.3. Median HR MAS NMR Spectra Obtained for Different Tissue Components

**Figure 1:**
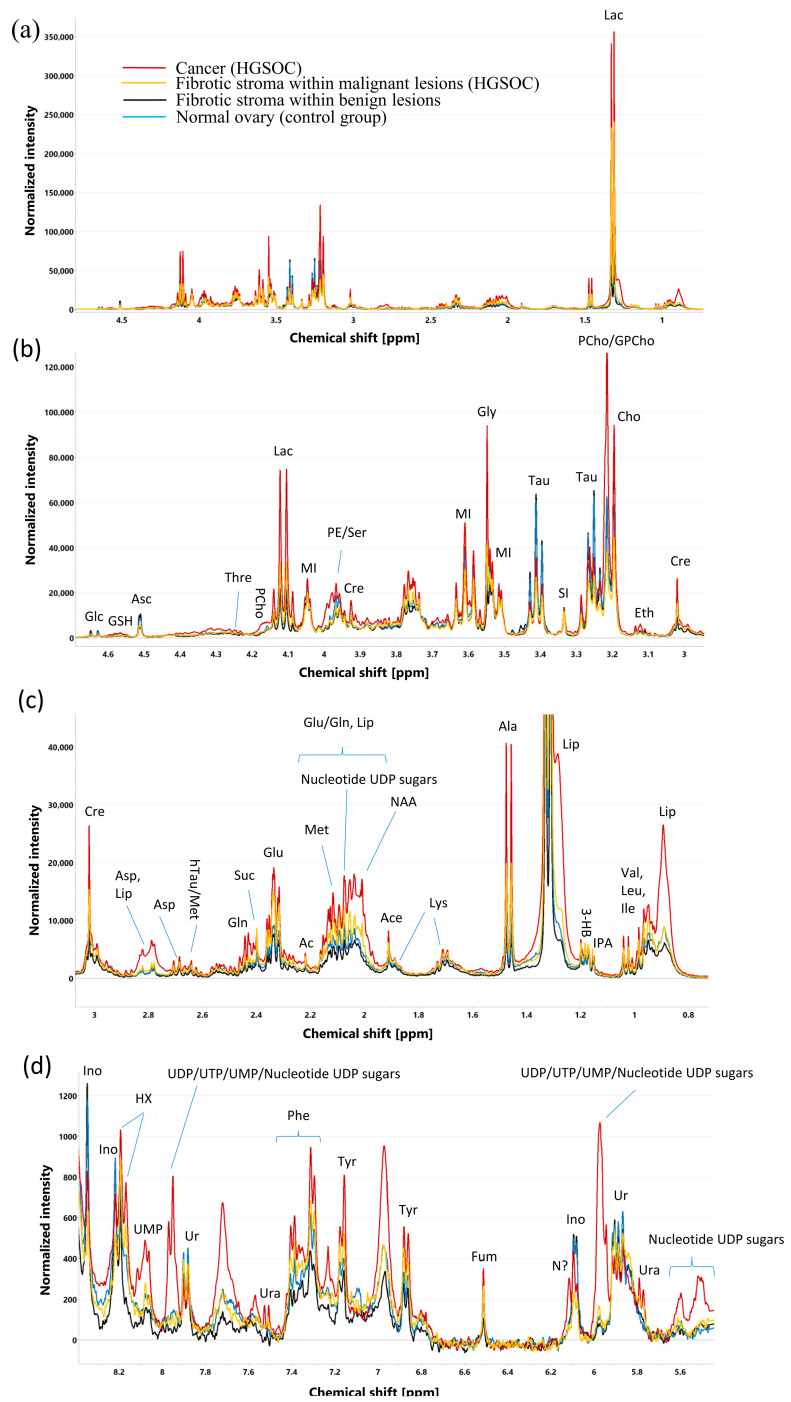
Median ^1^H CPMG HR-MAS NMR spectra [region 0.7–4.8 ppm (**a**), region 2.9–4.8 ppm (**b**), region 0.7–3.1 ppm (**c**), and region 5.5–8.4 ppm (**d**)] of the cancer compartment, fibrotic compartment within malignant tumors, fibrotic compartment within benign tumors, and normal ovary tissue (samples collected from control group). Assignment of signals: Lip—lipids, Val—valine, Ile—isoleucine, Leu—leucine, IPA—isopropanol, 3-HB—3-hydroxybutyrate, Lac—lactate, Ala—alanine, Lys—lysine, Ace—acetate, NAA—N-acetylaspartate, Glu—glutamate, Gln—glutamine, Met—methionine, Ac—acetone, Suc—succinate, hTau—hipotaurine, Asp—aspartate, Cre—creatine, Eth—ethanolamine, Cho—choline, PCho—phosphocholine, GPCho—glycerophosphocholine, SI—scylloinositol, Tau—tauryna, Gly—glycine, MI—Myo-inositol, PE—phosphoethanolamine, Ser—serine, Asc—ascorbate, Thre—threonine, GSH—glutathione, Glc—glucose, Ura—uracil, Ur—uridine, UDP—uridine-5′-diphosphate, UTP—Uridine-5′-triphosphate, UMP—Uridine 5′-monophosphate, Ino—inosine, Fum—fumarate, Tyr—tyrosine, Phe—phenylalanine, HX—hypoxantine, N?—unassigned signal.

The median CPMG (Carr–Purcell–Meiboom–Gill) spectra obtained for the samples that are representative for HGSOC, fibrotic stroma within malignant tumors, fibrotic stroma within benign tumors, and normal ovary tissue are shown in Figure 1, while the median p-Jres spectra (one-dimensional projections of 2D J-resolved spectra onto the chemical shift axis) for these tissue components can be found in Figure S3.

Most of the detected metabolites were visible in both the CPMG and p-Jres spectra. However, the levels of BCAA (branched chain amino acids: valine, leucine, and isoleucine), N-acetylaspartate, and the singlet at 2.07 ppm (assigned to uridine diphosphate nucleotide sugars) were evaluated solely in the p-Jres spectra due to a lower signal overlap with lipids, whereas the levels of lysine, glutathione, and the signals in the aromatic region were quantitated in the CPMG spectra only.

### 2.4. Multivariate Analysis

**Figure 2:**
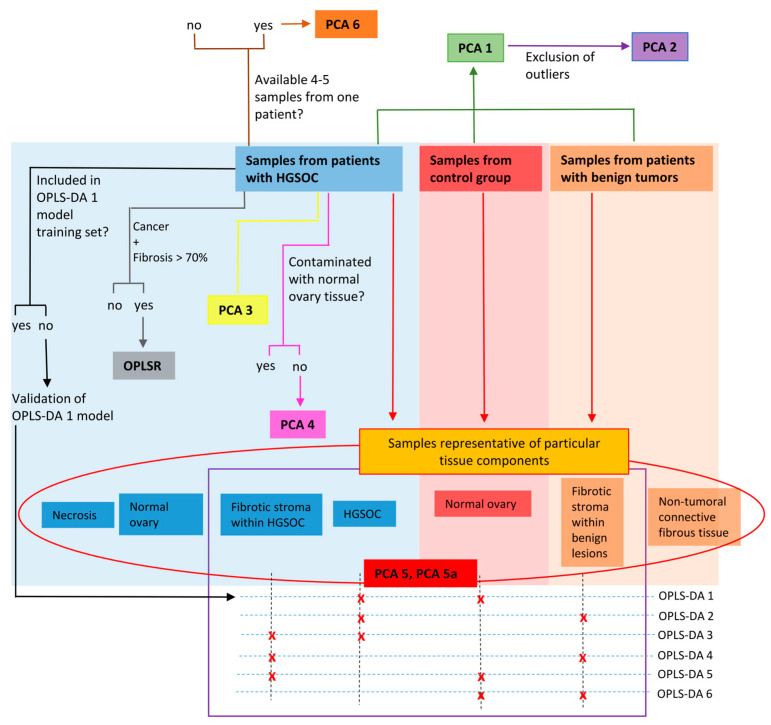
The scheme of the multivariate analysis. PCA—principal component analysis, OPLSR—orthogonal partial least squares regression, OPLS-DA—orthogonal partial least squares discriminant analysis.

The multivariate analysis scheme is presented in Figure 2.

The first step was an unsupervised exploration of the intrinsic clustering of all the collected samples using principal component analysis (PCA). This analysis was performed based on the CPMG spectra.

The initial PCA model 1 revealed eight outliers (seven samples excised from the cancer patients and one sample from a patient with benign tumor) that were removed before the final analyses. The large lipid signals were found to be responsible for the detected outlyingness. To minimize the influence of the residual lipid peaks, the regions: 0–1.41 ppm, 1.51–1.67 ppm, 1.94–2.29 ppm, 2.72–2.93 ppm were excluded from the subsequent analyses. All remaining spectra obtained for the patients diagnosed with malignant tumors, benign tumors, and from the control patients were subjected to PCA model 2. The samples collected from the patients diagnosed with ovarian cancer only were included in the dataset used for the construction of PCA model 3, while the metabolic profiles measured from the malignant tumor samples not contaminated with normal ovary tissue were used for the development of PCA model 4. Finally, the spectra obtained from the specimens that were representative of the particular tissue components (cancer, fibrosis, normal ovary, etc.) were included in PCA model 5.

To avoid overrepresentation of patients for whom the spectra from several neighboring regions were available, the number of samples per patient was minimized in PCA model 5a. The samples representative of cancer were chosen based on the highest fraction of cancer cells. However, if two specimens from the same patient were found to be representative of different tumor components (i.e., cancer and fibrotic stroma), they were both included in the analyses. The samples collected bilaterally were considered as independent samples.

The number of the samples that were representative of particular tissue components and the number of the samples included in PCA model 5a are given in Table 4. In total, 55 samples from 50 patients were included in the PCA 5a model.

The spectra that make up the ‘heterogeneity dataset’ were subjected to PCA model 6 for visual assessment of intra-tumor and inter-patient metabolic heterogeneity. The samples characterized with a high necrosis fraction (above 30%) and/or contaminated with normal ovarian tissue were excluded from the analysis.

The metabolic differences between the samples that are representative of particular tissue components (included in PCA model 5a) were also studied in detail using a supervised orthogonal partial least squares discriminant analysis (OPLS-DA) of the CPMG and p-Jres spectra (the type of the spectra used in the analysis is indicated with a subscript CPMG or J-res in the model name). The OPLS-DA models built to distinguish between various tissue components are collected in Table 5.

Post-HR MAS NMR histological verification of the samples excised from the patients with ovarian cancer showed that some of them were contaminated with normal ovarian tissue. All these samples (not included in the training sets of the OPLS-DA 1_CPMG_ model and the OPLS-DA 1_J-res_ model) were used as a test set to assess the predictive ability of these models.

The relation between a metabolic profile (X data matrix) and a percentage of cancer cells (Y variable) was analyzed using an orthogonal partial least squares regression (OPLSR) method. The samples collected from malignant tumors containing mainly cancer cells and fibrotic stroma (the total contribution of these two components was required to be greater than 70%) were included in this analysis. When several samples from the same patient met this criterion, the sample with the highest cancer cell fraction was chosen for the development of the model.

### 2.5. Unsupervised Analysis—PCA Models 2,3,4,5,5a

**Figure 3:**
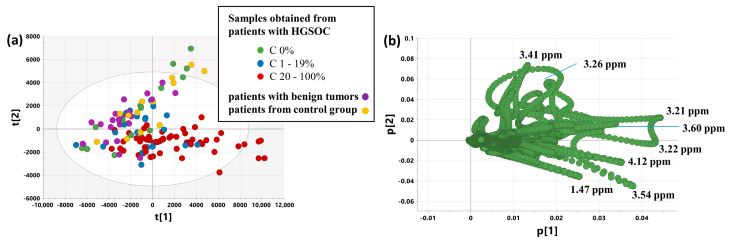
The scores (**a**) and loadings (**b**) plots obtained from the PCA model 2. The presented projection plane represents 57.6% of the total variation in the dataset. The scores and loadings for the i-th principal component are denoted as t[i] and p[i]. C 0%—the samples containing no cancer cells, C 1–19%—the samples characterized by a cancer content of 1–19%, C 20–100%—the samples characterized by a cancer content of 20–100%.

Figure 3 presents the scores and loading plots for the first two principal components obtained from the PCA analysis (model 2) of the samples collected from the patients with malignant and benign tumors and from the control group. The samples collected from the cancer patients were colored according to the fraction of cancer cells. As seen in Figure 3a, the fourth quadrant is occupied mainly by the C 20–100% samples, whereas the second and third ones are occupied by the samples excised from the control group, the patients with nonmalignant lesions, and the C 0% samples. The samples obtained from malignant tumors and characterized with a fraction of cancer cells from 1 to 19% are located around the origin of the principal component system.

The loadings plot (Figure 3b) indicates that the major variance direction is dominated by the positive contributions of phosphocholine/glycerophosphocholine (3.21 ppm, 3.22 ppm), lactate (4.12 ppm), glycine (3.54 ppm), myo-inositol (3.60 ppm), and alanine (1.47 ppm), and the second variance direction has positive contributions from the taurine signals (3.41 and 3.26 ppm).

**Figure 4:**
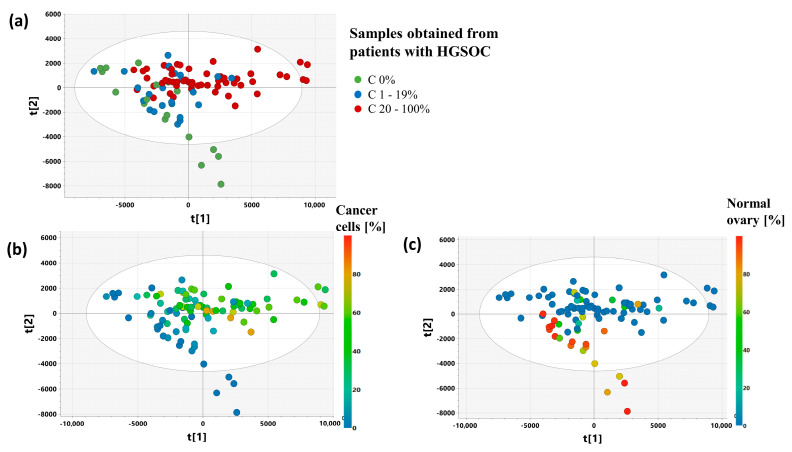
The scores plot obtained from PCA model 3 presenting the distribution of the samples belonging to the following groups: C 0% (the samples containing no cancer cells), C 1–19% (the samples characterized by a cancer content of 20–100%), C 20–100% (the samples characterized by a cancer content of 20–100%) (**a**) and colored according to a cancer cell fraction (**b**) and a normal ovary tissue fraction (**c**). The presented projection plane represents 55.6% of the total variation in the dataset. The scores for the i-th principal component are denoted as t[i].

The PCA 3 model computed based on the samples obtained from the cancer patients only also shows that the first principal component is associated with a cancer cell fraction, while the second one is associated with a considerable admixture of normal ovary tissue (Figure 4).

**Figure 5:**
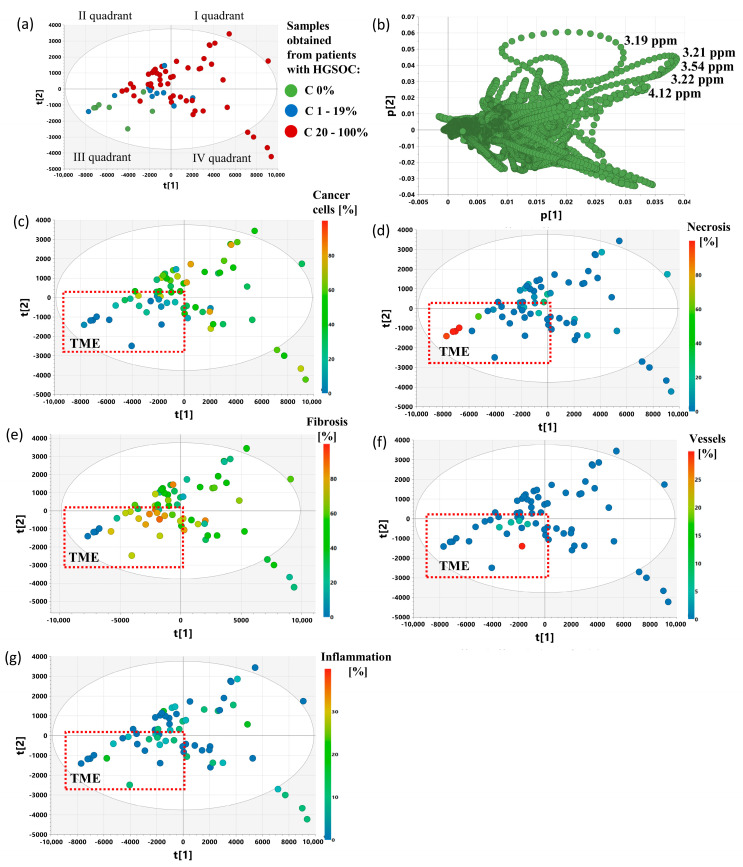
The scores plot obtained from PCA model 4 presenting the distribution of the samples belonging to the following groups: C 0% (the samples containing no cancer cells), C 1–19% (the samples characterized by a cancer content of 20–100%), C 20–100% (the samples characterized by a cancer content of 20–100%) (**a**) and colored according to the fraction of cancer cells (**c**) necrosis (**d**), fibrosis (**e**), vessels (**f**), and inflammation (**g**). The loadings plot obtained from PCA model 4 (**b**). The presented projection plane represents 62.7% of the total variation in the dataset. The scores for the i-th principal component are denoted as t[i]. TME—tumor microenvironment.

The scores plot obtained after the exclusion of the samples contaminated with normal ovarian tissue (PCA model 4, Figure 5) shows that the cancer samples tend to be separated from the samples characterized by the high contents of certain components of the tumor microenvironment (such as fibrosis, necrosis, and vessels). The latter samples are located in the third quadrant of the principal component system (Figure 5c–f). Their position (Figure 5b) is mainly influenced by relatively low levels of phosphocholine and glycerophosphocholine (3.21 and 3.22 ppm), choline (3.19 ppm), glycine (3.54 ppm), and lactate (4.12 ppm). It is noteworthy that the samples containing immune cells are distributed fairly uniformly in the principal component space. This could be due to a relatively low content of these cells in the studied samples (<15%), as revealed by histopathological verification.

**Figure 6:**
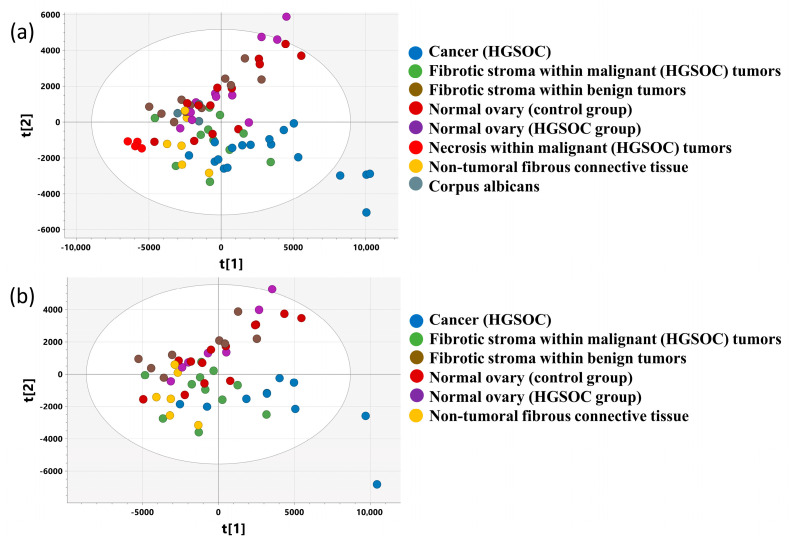
The scores plot obtained from (**a**) PCA model 5 including all the samples fulfilling the criteria presented in Table 3 and (**b**) PCA model 5a computed based on a dataset reduced in order to limit the number of the samples per patient. The scores for the i-th principal component are denoted as t[i].

Finally, the CPMG spectra obtained from the samples that are representative of particular tissue types were subjected to PCA model 5. The scores plot for the first two principal components shows that the cancer samples are separated from the normal ovary tissue (excised from both the control group and the cancer patients) (Figure 6a). A trend towards separation of the cancer samples from the samples containing mainly fibrotic stroma and necrosis within the malignant lesions can be observed along the first principal component. Interestingly, the fibrotic stroma within the malignant lesions has been shown to occupy an area different from that of the intratumoral fibrosis within the benign lesions. The latter tissue type cannot be distinguished from the normal ovarian tissue, but it is quite well separated from the extra-tumoral connective tissue samples collected from the patients with benign tumors.

The scores plot obtained from the model analogous to the previous one but computed based on a reduced dataset to limit the number of samples analyzed per patient is shown in Figure 6b.

### 2.6. OPLS-DA 1_CPMG_ and OPLS-DA 2_CPMG_ Models

**Figure 7:**
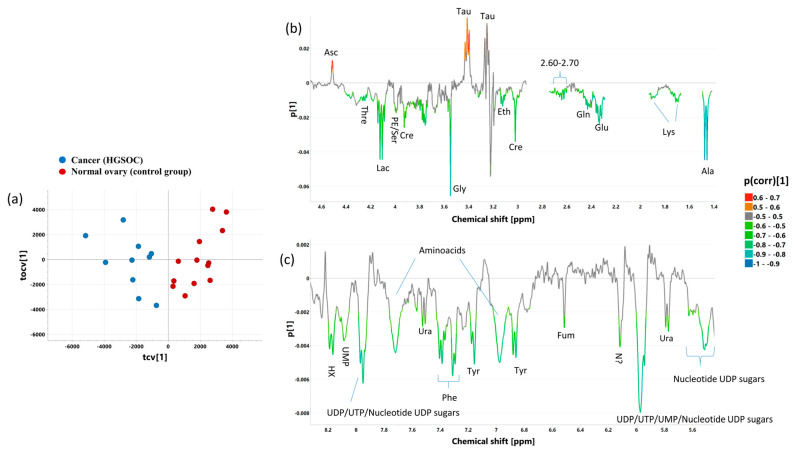
The cross-validated scores (**a**) and loading plots [aliphatic region—(**b**), aromatic region—(**c**)] obtained from the OPLS-DA 1_CPMG_ model. The cross-validated scores for the predictive component are denoted as tcv[1], whereas for the orthogonal one, they are designated as tocv[1]. The loadings for the predictive component are denoted as p[1]. The signals in the loadings plots are colored according to the p(corr)[1] values (the loadings scaled as correlation coefficients between the original data and the scores obtained for the first component). The abbreviations for metabolites are the same as in the legend of Figure 1.

**Figure 8:**
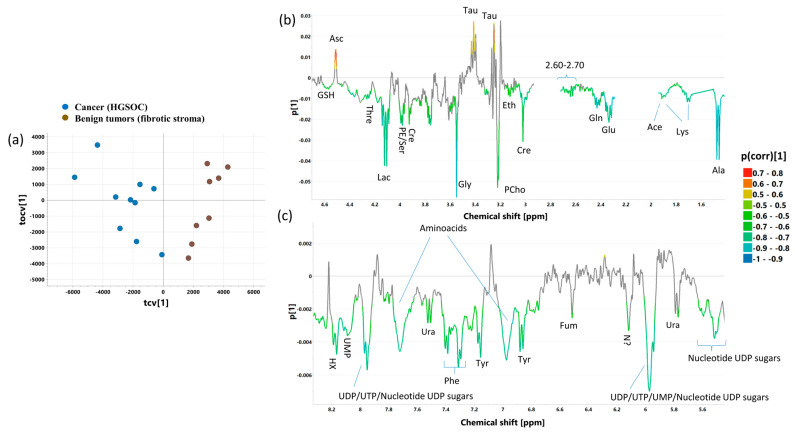
The cross-validated scores (**a**) and loadings plots [aliphatic region—(**b**), aromatic region—(**c**)] obtained from OPLS-DA 2_CPMG_ model. The cross-validated scores for the predictive component are denoted as tcv[1], while for the orthogonal one, they are denoted as tocv[1]. The loadings for the predictive component are denoted as p[1]. The signals in the loadings plots are colored according to the p(corr)[1] values (the loadings scaled as correlation coefficients between the original data and the scores obtained for the first component). The abbreviations for metabolites are the same as in the legend of Figure 1.

The cross-validated scores and loading plots obtained from the OPLS-DA 1_CPMG_ model (R2 = 85.8%, Q2 = 74%, p CV-ANOVA = 0.000042) differentiating cancer from normal ovary tissue and from the OPLS-DA 2_CPMG_ model (R2 = 92.3%, Q2 = 74.4%, p CV-ANOVA = 0.00083) discriminating cancer from the benign lesions are presented in Figure 7 and Figure 8, respectively. The p(corr)[1] values (loadings scaled as correlation coefficients between the original data and scores obtained for the first component), the variable influence at projection (VIP) values for both models, and the results of the univariate statistical analysis (the fold-changes and the *p* values) for the metabolites contributing to the class separation are listed in Table S1. A good separation between the studied groups in the scores plots is apparent (Figure 7a and Figure 8a, for the OPLS-DA 1_CPMG_ model and the OPLS-DA 2_CPMG_ model, respectively). The loadings plots (Figure 7b,c and Figure 8b,c showing the corresponding aliphatic and aromatic ranges of the chemical shifts) indicate that the most important differentiating features are common in both models. They are as follows:

- There are higher levels of taurine and ascorbate in the normal ovary tissue and in the benign tumors than in the cancer samples.
- There is less alanine, lysine, glutamine, and glutamate, and lower intensity of overlapping signals in the region from 2.60 to 2.70 ppm (arising from hypotaurine, methionine, NAA, and aspartate), as well as less creatine, ethanolamine, glycine, phosphoethanolamine/serine, lactate, threonine, fumarate, tyrosine, phenylalanine, uracil, hypoxantine, uridine nucleotides (UTP/UDP/UMP), and associated nucleotide sugars, and lower intensity of the unknown signal at 6.11 ppm in the normal ovary and in the benign tumors than in cancer.
- Lower glutathione, phosphopcholine, acetate contents and lower intensity of the signals at 3.97 and 3.98 ppm were found in the benign lesions than in cancer samples, (it should be mentioned that the values of p(corr)[1] for acetate and phosphocholine in the model differentiating normal ovary tissue and cancer are only slightly lower than 0.5).

As seen from the analysis of the VIP values, the contribution of the aliphatic region to the abovementioned OPLS-DA models is higher than the contribution of the aromatic area, although in the OPLS-DA 1_CPMG_ model the VIP value above 1 identifies the aromatic signal at 5.97 ppm as being markedly altered.

Univariate analysis confirms the following (Table S1):

- Lower alanine, glutamine, glutamate, creatine, glycine, lactate, hypoxantine, uridine nucleotides (UTP/UDP/UMP), and nucleotide UDP sugars in the normal ovary and in the benign tumors than in cancer,
- Lower lysine, threonine, tyrosine, phenylalanine, acetate, glutathione, and phosphoethanolamine/serine in the benign tumors with respect to cancer.
- Lower level of ethanolamine, a downward trend of acetate, and an upward trend of ascorbate in the normal ovarian tissue with respect to cancer.

### 2.7. OPLS-DA 1_J-res_ and OPLS-DA 2_J-res_ Models

The cross-validated scores and loading plots obtained from the OPLS-DA 1_J-res_ model (R2 = 87.3%, Q2 = 75.9%, p CV-ANOVA = 0.000022) differentiating the tissue samples of cancer and normal ovary and the OPLS-DA 2_J-res_ model (R2 = 89.8%, Q2 = 71.9%, p CV-ANOVA = 0.00148) discriminating the cancer from the benign lesions are presented in Figures S4 and S5, respectively.

Like the models built on the CPMG spectra, the OPLS-DA 1_J-res_ and OPLS-DA 2_J-res_ models show higher taurine and ascorbate and lower alanine, lactate, acetate, glutamate, glutamine, glycine, creatine, phosphoethanolamine/serine, hypotaurine, and threonine in the normal ovary and benign tumors compared to in the cancer samples (Table S2). The p(corr)[1] value for phosphocholine is greater than 0.5 in the OPLS-DA 1_2-res_ model and slightly lower than 0.5 in the OPLS-DA 1_J-res_ model (as for the CPMG models). A lower level of ethanolamine and succinate was detected in the normal ovary compared to cancer.

Furthermore, the analysis of the J-resolved spectra revealed the spectral changes in the regions excluded from the analysis of the CPMG spectra involving branched chain amino acids (isoleucine, valine, and leucine), N-acetylaspartate, and UDP sugars (2.07 ppm). These signals were found to be lower in both the normal and benign samples than in the cancer ones.

The results obtained from the OPLS-DA1_J-res_ model are in a general agreement with those received from the univariate analysis. However, the changes in the integral signal intensities of taurine, ascorbate, phosphocholine, phosphoethanolamine/serine, acetate, and succinate did not reach statistical significance in the univariate analysis.

### 2.8. Assessment of the Predictive Ability of the OPLSDA Models Differentiating Cancer from Normal Ovary

**Figure 9:**
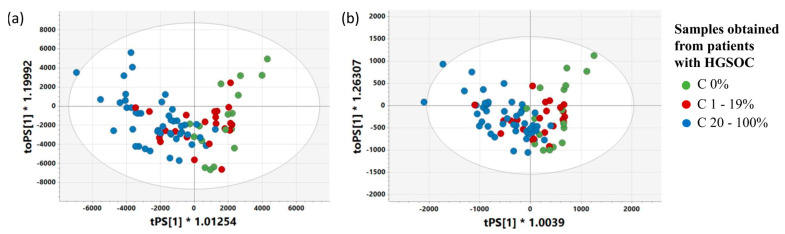
The predicted scores plots obtained from the OPLS-DA 1_CPMG_ (**a**) and OPLS-DA 1_J-res_ (**b**) models. The predicted scores plots for the predictive and orthogonal components are denoted as tPS[1] and toPS[1], respectively. C 0%—the samples containing no cancer cells, C 1–19%—the samples characterized by a cancer content of 1–19%, and C 20–100%—the samples characterized by a cancer content of 20–100%.

The predictive ability of the OPLS-DA 1_CPMG_ and CPMG 1_J-res_ models was evaluated using a test set consisting of all the samples collected from patients with ovarian cancer not included in the training set. Figure 9 shows the predicted scores plots. Assuming that the cancer samples are assigned the negative PC1 scores, whereas the normal ovary tissue samples are assigned the positive ones, the classification accuracy of the OPLS-DA 1_CPMG_ model is 90% (sensitivity 91%, specificity 88%) for the samples characterized by the percentage of cancer cells equal to or above 20%. The analogous parameters for CPMG 1_J-res_ are as follows: classification accuracy of 81% (sensitivity 80%, specificity 94%). The samples characterized by the percentage of cancer cells from 1 to 20% are not well classified by the models. This can be explained by the significant contribution of normal ovarian tissue to the total volume in these samples.

### 2.9. OPLS-DA 3_CPMG_ and OPLS-DA 3_J-res_ Models

**Figure 10:**
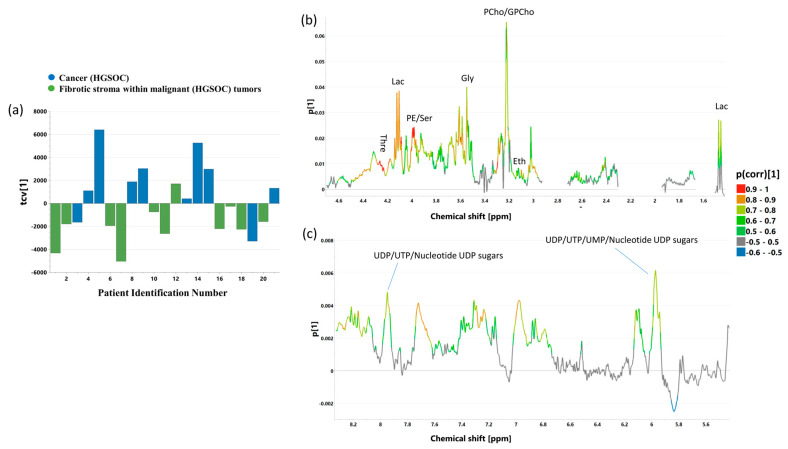
The cross-validated scores (tcv[1]) (**a**) and loading (p[1]) plots [aliphatic region—(**b**), aromatic region—(**c**)] obtained from the OPLS-DA 3_CPMG_ model. The signals in the loadings plots are colored according to the p(corr)[1] values (the loadings scaled as correlation coefficients between the original data and the scores obtained for the first component). The abbreviations for metabolites are the same as in the legend of Figure 1.

The OPLS-DA technique applied to the CPMG and p-Jres spectra for discrimination between cancer and fibrotic stroma resulted in the one-component models [OPLS-DA 3_CPMG_: R2 = 44.7%, Q2 = 32.5%, p CV-ANOVA = 0.02917; OPLS-DA 3_J-res_: R2 = 55.8%, Q2 = 49.1%, p CV-ANOVA = 0.0023]. The scores and loading plots obtained from these models are presented in Figure 10 and Figure S6, respectively. For both models, the loadings plots show that in the cancer samples, the majority of the signals are larger than in those containing mainly intratumoral fibrosis. Indeed, the fold changes (computed as ratios of the median metabolite levels in the cancer specimens to the median metabolite levels in fibrosis) are greater than 1 for the majority of the evaluated signals. The exceptions to this rule are, inter alia, the signals of glucose, ascorbate in both types of the spectra, uridine (5.88, 5.79 ppm) and uracil (5.88 ppm) in the CPMG spectra, and acetone (2.22 ppm) in the p-Jres spectra. These metabolites are characterized by |p(corr)[1]| < 0.25 in the developed OPLS-DA models.

However, the univariate analysis of the spectral integrals obtained from the CPMG spectra confirms only the following:

- Increased phosphoethanolamine/serine, phosphocholine/glycerophosphocholine, threonine, and nucleotides derived from uridine (UDP and/or UTP) in the samples representative of cancer compared to those reflecting fibrotic stroma,
- The upward trends in lactate, glycine, and ethanolamine in the samples characterized by a high percentage of cancer cells compared to those containing mainly fibrosis.

These metabolites have been marked in the loadings plot obtained from the OPLS-DA 3_CPMG_ model (Figure 10), while the corresponding p(corr)[1] and VIP values for these metabolites are shown in Table S3.

Significant changes (or trends) in the levels of phosphoethanolamine/serine, phosphocholine/glicerophosphocholine, threonine, and lactate were also received from univariate analysis of the spectral integrals determined from the p-Jres spectra (Table S4). This analysis also revealed that, in the cancerous samples, the signals corresponding to UDP sugars (2.07 ppm) and glutamine are significantly higher than in the fibrotic stroma samples, whereas for valine and creatine increasing trends are observed.

### 2.10. OPLS-DA 4_CPMG_ and OPLS-DA 4_J-res_ Models

**Figure 11:**
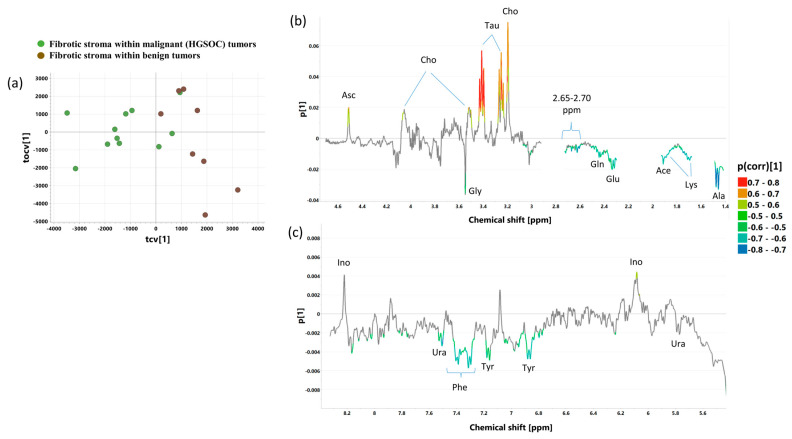
The cross-validated scores (**a**) and loading plots [aliphatic region—(**b**), aromatic region—(**c**)] obtained from the OPLS-DA 4_J-Res_ model. The cross-validated scores for the predictive component are denoted as tcv[1], whereas for the orthogonal one, they are denoted as tocv[1]. The loadings for the predictive component are denoted as p[1]. The signals in the loadings plots are colored according to the p(corr)[1] values (the loadings scaled as correlation coefficients between the original data and the scores obtained for the first component). The abbreviations for metabolites are the same as in the legend of Figure 1.

The cross-validated scores and loading plots of the OPLS-DA 4_CPMG_ (R2 = 70.5%, Q2 = 53.9%, p CV-ANOVA = 0.02110) and OPLS-DA 4_J-res_ (R2 = 75.5%, Q2 = 54.1%, p CV-ANOVA = 0.02054) models discriminating the fibrotic compartment within malignant tumors from the fibrotic compartment within benign lesions are presented in Figure 11 and Figure S7, respectively. The p(corr)[1] and VIP values obtained from the multivariate analysis and the results from the univariate statistical evaluation (fold-changes and *p* values) for the metabolites important for the class separation are listed in Tables S5 and S6. The OPLS-DA 1_CPMG_ model revealed that within the benign lesions, the samples representative of the fibrous stroma show higher taurine, ascorbate, choline, and inosine, and lower alanine, lysine, acetate, and metabolites contributing to the spectral region of 2.60 to 2.70 ppm, as well as glutamine, glutamate, glycine, tyrosine, phenylalanine, and uracil (7.53 ppm) compared to the fibrotic stroma of the malignant tumor. The changes in the levels of glutamine, glutamate, acetate, lysine, and alanine were found to be statistically significant in the univariate analysis (Table S5).

The loadings plot obtained from the OPLS-DA 4_J-res_ model also shows that the fibrotic stroma within malignant tumors and the fibrotic stroma within benign tumors differ metabolically (which is also visible in the CPMG spectra). In the fibrotic stroma within malignant tumors, alanine, acetate, glutamine, glutamate, glycine, the branched chain amin acids (leucine, isoleucine, valine), and threonine are higher, whereas taurine and ascorbate are lower than in the fibrotic stroma within benign tumors. However, a univariate analysis of the spectral integrals determined from the p-Jres spectra confirms only the significance of the changes in the levels of leucine and isoleucine (Table S6).

### 2.11. OPLS-DA 5_CPMG_, OPLS-DA 5_J-res_, OPLS-DA 6_CPMG_, and OPLS-DA 6_J-res_ Models

The multivariate OPLS-DA models constructed to differentiate benign tumors (composed mainly of fibrosis) from normal ovary tissue (OPLS-DA 5_CPMG_, OPLS-DA 5_J-res_) and fibrotic stroma within malignant tumors from normal ovary tissue (OPLS-DA 6_CPMG_ and OPLS-DA 6_J-res_) were not statistically significant according to cross-validation.

### 2.12. OPLSR Regression

**Figure 12:**
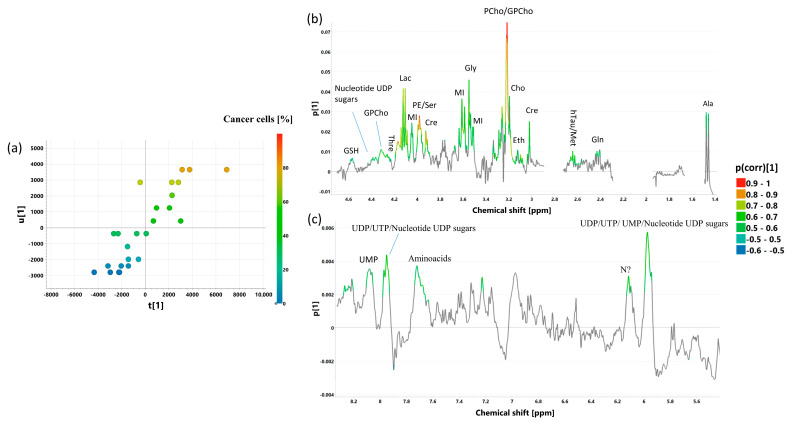
The scores (**a**) and loadings plots [aliphatic region—(**b**), aromatic region—(**c**)] obtained from the OPLSR _CPMG_ model. The X-scores for the predictive component are denoted as t[1], whereas the Y-scores are denoted as u[1]. The loadings for the first predictive component are denoted as p[1]. The signals in the loadings plots are colored according to the p(corr)[1] values (the loadings scaled as correlation coefficients between the original data and the scores obtained for the first component). The abbreviations for metabolites are the same as in the legend of Figure 1.

The samples composed mainly of cancer cells and fibrotic stroma (a total amount of these two components above 70%) were subjected to OPLS analysis to find the relationship between histological composition and metabolic profile. The developed model was characterized by a low prediction ability. However, after the exclusion of seven samples containing necrotic areas, good quality models were obtained based on the CPMG (OPLSR _CPMG_: R2 = 74.7%, Q2 = 56%, p CV-ANOVA = 0.00058) and J-resolved (OPLSR _J-res_, R2 = 82%, Q2 = 55.1%, p CV-ANOVA = 0.0022) spectral data. The results obtained from these models are presented in Figure 12 and Figure S8, respectively.

**Figure 13:**
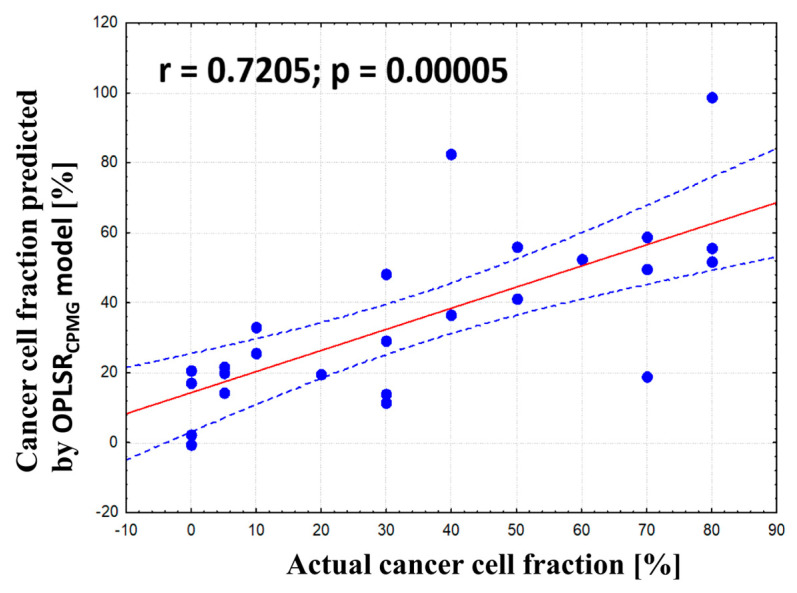
The cancer cells fraction predicted by the OPLS_CPMG_ model (based on cross-validation) vs. the actual cancer content determined from histological examinations. r—Pearson correlation coefficient.

The OPLSR models built on the CPMG and J-resolved spectra indicate that the fraction of cancer cells is positively correlated to the levels of lactate, myo-inositol, phosphoethanolamine/serine, phosphocholine/glycerophosphocholine, ethanolamine, glutamine, and hypotaurine. For these metabolites, the values of p(corr)[1] are > 0.5 (Tables S7 and S8). The signals of glutathione, glycine, creatine, and threonine were also assigned p(corr)[1] > 0.5 in the analysis of the CPMG spectra and the values of 0.4 to 0.5 in the analysis of the J-resolved spectra. Moreover, the loadings plot from the OPLS-DA 1_CPMG_ model indicates a positive relationship between cancer content and the levels of alanine, choline, and nucleotides derived from uridine (UDP/UTP/UMP) and/or associated nucleotide sugars, whereas the OPLS-DA 1_J-res_ model shows that the higher the cancer content, the higher the intensities of the signals at 2.01 ppm (NAA) and 2.07 ppm (UDP sugars). As seen in Figure 13 and Figure S9, the histologically verified percentage content of the transformed cells and the tumor purity predicted by the model (using seven-fold cross-validation) are in a general agreement (r = 0.72 for the CPMG data and r = 0.74 for the p-Jres data).

The relationships between tumor purity and integral signal intensities derived from the CPMG and p-Jres spectra were also studied using univariate regression analysis (the results are in Table 6 and Table S9, respectively). Strong positive associations are evident for both types of the spectra between the fraction of cancer cells and the total level of phosphocholine and glycerophosphocholine (r > 0.7) and for the phosphocholine level determined using an automatic deconvolution of the CPMG spectra (Table 6). The glutamine level is also found to correlate strongly with tumor purity in the p-Jres spectra (r > 0.7, Table S9), while the appropriate correlation coefficient obtained from the CPMG spectra is slightly lower (r = 0.65). Furthermore, moderate associations (0.5 < r < 0.7) are also found between a fraction of cancer cells and lactate (both in the CPMG and p-Jres spectra), phosphoethanolamine/serine (both in the CPMG and p-Jres spectra), the UDP/UTP/UMP nucleotide signals and glycerophosphocholine (in the CPMG spectra), and UDP sugars (at 2.07 ppm) and succinate (in the p-Jres spectra). For completeness, the metabolites that have weak correlations (0.3 < r < 0.5) with the percentage of cancer are also included in Table 6 and Table S9.

### 2.13. Joint Univariate and Multivariate Analysis

The results obtained from joint univariate and multivariate analysis are summarized in Table 7.

### 2.14. PCA Model 6

The intra-tumoral and inter-patient metabolic variabilities were analyzed based on the ‘heterogeneity dataset’ consisting of 34 CPMG spectra acquired from the tissue samples excised from 8 patients for whom 4–5 neighboring specimens were available. The fractions of cancer cells and fibrosis in the studied samples calculated for each patient are presented in Figure S10. The median cancer content ranges from 35 to 70%, while the content of fibrotic stroma varies from 30 to 50%. The Kruskal–Wallis test did not show statistically significant differences in the fractions of these two major tissue components in the samples collected from different patients (*p* > 0.05). The percentage of necrosis content in the analyzed samples was below 30%, while the contribution of inflammation was below 10%.

**Figure 14:**
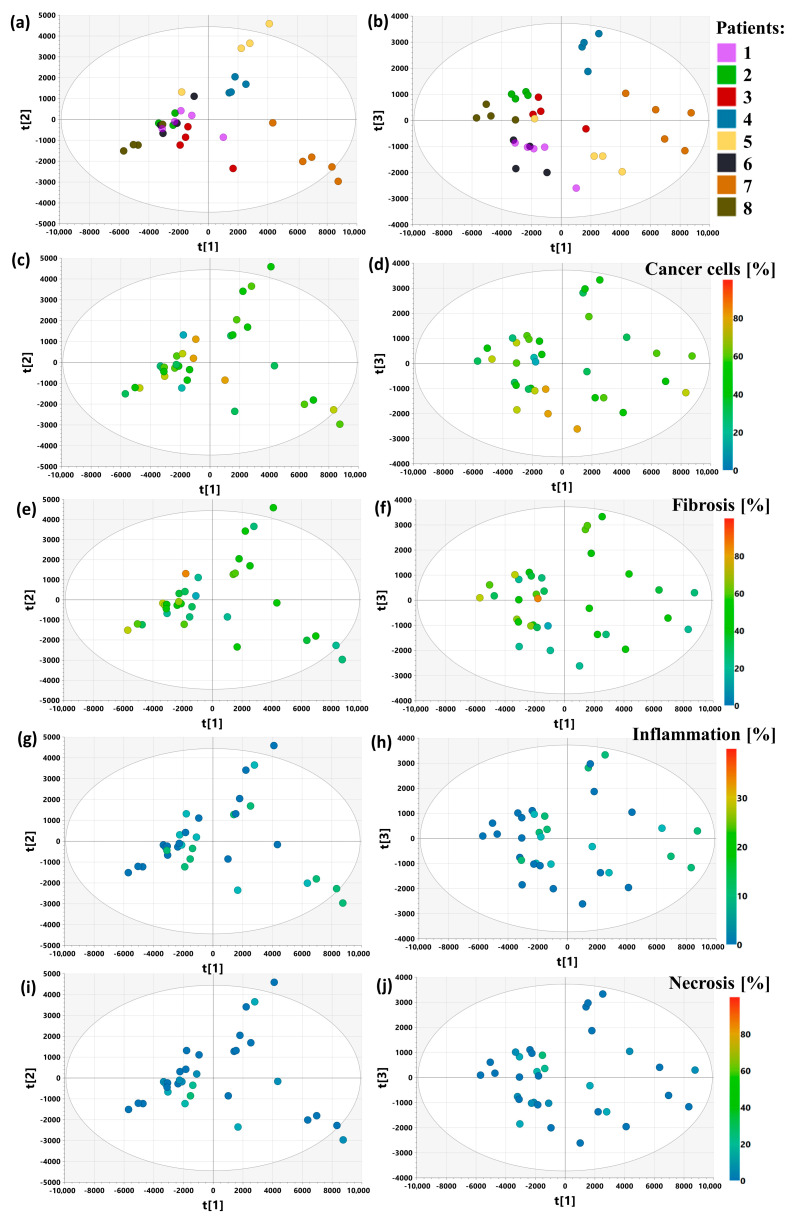
The scores plots obtained from the PCA analysis of the CPMG spectra acquired from the samples forming the ‘heterogeneity datasets’ (PCA model 6): t[1] vs. t[2] scores colored according to the patient identification number (**a**), cancer cells content (**c**), fibrosis fraction (**e**), inflammation fraction (**g**), necrosis fraction (**i**); t[1] vs. t[3] scores colored according to the patient identification number (**b**), cancer cells content (**d**), fibrosis fraction (**f**), inflammation fraction (**h**), necrosis fraction (**j**); the coordinate system formed by the first three principal components explains 73% of the total variation in the dataset. The scores for the i-th principal component are denoted as t[i].

The principal component analysis (Figure 14) shows that in the evaluated dataset, the inter-individual variation is higher than the intra-individual variation. The combination of the histological data and the results of the metabolic profile analysis indicates that the natural clustering of the samples seen in the scoring plot cannot be explained by the histological content of the samples.

## 3. Discussion

HGSOC is the most lethal gynecologic malignancy, with a 5-year survival rate of less than 35% for patients diagnosed in advanced stages [23]. The identification of novel diagnostic and predictive biomarkers is hampered by spatial and temporal molecular heterogeneity—a vital characteristic of this cancer. Despite substantial efforts to understand the complexity of HGSOC at the genomic, transcriptomic, and proteomic levels [24,25], the translation of research results into clinical practice is limited. Metabolomics has been less explored, so far, though it seems to be very promising among the ‘omics’ methods. It deals with small-molecule compounds (≤1.5 kDa) and is considered to be the closest to cancer phenotypes. Importantly, metabolic abnormalities in tumors are not always related to genetic ones [26].

In recent years, the HR MAS NMR technique has been increasingly used for the identification of clinically relevant cancer subtypes [27,28]. The non-invasive nature of the technique substantially reduces the confounding effect of varying contribution of different tumor components in the interpretation of the metabolic properties of tumor samples. The impact of intra-tumoral heterogeneity on HR MAS NMR-derived metabolic profiles was studied in detail in breast cancer [29,30,31]. However, there is a paucity of similar studies regarding HGSOC. In our work, the HR MAS NMR analysis of the tissue samples excised from ovarian lesions and normal ovary was coupled to the standard histological analysis to investigate metabolic features of malignancy with a special focus on the relation between the metabolic profile and tumor purity. As the importance of desmoplastic tumor milieu and molecular interaction between cancer-associated fibroblasts (CAFs) and cancer cells in tumor progression has been shown in many works [32,33], we also aimed to compare the metabolic profiles of the fibrotic stroma in the benign and malignant tumors.

The surgical excisional biopsy specimens for research purposes are frequently collected based on an intraoperative macroscopic examination of the pathological nature taking into account their color, stiffness and shape [34]. Therefore, the non-malignant tissue admixture is a commonly encountered problem. Multivariate analyses performed in our work showed that the contamination of tumor specimens with normal ovarian tissue hampers cancer detection in the low-purity samples (below 20%). A variable fraction of the cancer and stromal components in the samples excised from the HGSOC patients was also shown to constitute the source of considerable metabolic variability, in agreement with other works [35,36]. Although the tumor samples characterized by a high cancer fraction (>60%) could be easily differentiated from normal ovary tissue, it was found that the specimens collected from the benign tumors exhibit similar metabolic properties to normal ovary samples (PCA model 5a). It is well known that the main component of the benign tumors included in the PCA model 5a (serous cystadenofibromas and fibroma) is a stroma derived from the ovarian stroma [37]. Post-HR MAS NMR histopathological verification confirmed these findings. Interestingly, Zhong et al. reported that the benign tumors could be differentiated from the normal ovarian tissue in PCA scores plots obtained from the analysis of metabolic profiles derived from GC-MS. However, the histological content of the samples was not evaluated in their work [38].

The multivariate OPLS-DA models revealed that the only metabolites that exhibit lower levels in cancer tissue compared to normal ovary and benign tumors are ascorbate and taurine, compounds that play an important role in the female reproductive system [39,40]. Similar findings were reported in other studies [36]. However, the changes in taurine and ascorbate were not found to be significant in the univariate analysis in our work. Both types of analyses revealed that, in cancer, alanine, glutamine, glutamate, creatine, glycine, lactate, threonine, hypoxantine, hypotaurine, uridine nucleotides (UMP/UDP/UTP), nucleotide UDP sugars, NAA, and the branched chain amino acids are higher than in normal ovary samples and benign tumors. Lysine, tyrosine, phenylalanine, acetate, glutathione, and phosphoethanolamine/serine were found to be higher in cancer relative to the benign tumors, while ethanolamine was higher than in normal ovarian tissue.

Metabolic pathways (including glycolysis, amino acid metabolism, lipid metabolism, and the TCA cycle) are known to be deregulated in cancer cells to facilitate the uncontrolled proliferation and invasiveness [41]. Although many works report altered expression of glycolysis-related proteins (glucose transporter 1, GLUT1; hexokinase 2, HK2; lactate dehydrogenase A, LDHA) in ovarian cancer [42,43,44], our analysis did not reveal differences in the glucose levels between the studied groups of the samples. Garg et al. also found similar glucose concentrations in high-grade ovarian cancer and normal ovarian tissue [45]. However, the byproduct of glycolysis, lactate, was found to be higher in the malignant samples compared to the remaining ones in our work. The upregulation of L-type amino acid transporter 1 (LAT1) [46] and alanine serine cysteine transporter 2 (ASCT2) [47] observed in ovarian cancer could be related to the increase in the branched chain amino acids and glutamine in the malignant samples in our study. Glutamine is critically required for the survival of high-invasive ovarian cancer cells due to the multiple important roles it plays in the TCA cycle, anaplerosis, and the synthesis of macromolecules [48]. The conversion process of this amino acid to glutamate via glutaminase (glutaminolysis pathway) was also associated with the aggressiveness of cancer cells [49]. Glutamine participates in the synthesis of glutathione, an important antioxidant and reducing agent. An increase in glutamine, glutamate, and glutathione was observed in cisplatin-resistant A2780 ovarian cancer cells compared to those which were sensitive [50]. Of note, the A2780 human ovarian cancer cell line was established from an ovarian endometrioid adenocarcinoma tumor [51]. 3-phosphoglycerate and glutamate, provided by glycolysis and glutaminolysis, respectively, fuel the biosynthesis of serine, another compound essential for cancer cell survival. The three-step enzymatic process of serine production is catalyzed by 3-phosphoglycerate dehydrogenase (PHGDH), phosphoserine transaminase (PSAT), and serine phosphatase (PSPH). These enzymes are overexpressed in many cancer types, including ovarian cancer [52,53]. The synthesis of serine is closely related to the production of glycine via (serine hydroxymethyltransferase) SHMT. The upregulation of this protein in ovarian cancer [54,55] could be related to the higher level of glycine in the cancerous samples compared to the non-transformed tissue in our work.

Although the metabolic profiles of ovarian cancer, benign tumors, and/or normal ovary tissue were evaluated in several works using the HR MAS NMR technique [22] and mass spectrometry [34,35,36,56,57], the obtained results are inconsistent. Fong et al. revealed increased NAA, glutamine, lactate, fumarate, and uracil in ovarian cancer relative to normal ovarian tissue [57]. These changes are apparent in the multivariate analysis in our work, but the alterations of fumarate and uracil were not found to be significant in the univariate analysis. Zhong et al. [38] found a higher lactate level in cancer compared to both normal ovary and benign tumors, which is consistent with our results. However, glutamate, glutamine, glycine, and alanine were observed to be specifically higher in ovarian cancer than in the benign tumors, but not when compared to normal tissue. On the other hand, fumarate, serine, tyrosine, threonine, and lysine were found to be increased in the malignant samples relative to the non-involved ovary [38]. Recently, Oh et al. measured the amino acid and organic acid compositions of ovarian serous cancer, borderline, and benign tumors [58]. Several changes agree with our results (increased glutamine, creatine, lactate, and glycine in cancer), but they also found decreased levels of BCAA, lysine, tyrosine, and phenylalamnine in the malignant samples compared to the benign tumors. Zand et al. [56] showed an increase in NAA and ethanolamine in ovarian cancer compared to the non-involved ovarian tissue. The apparent inconsistency of the results could be attributed to the differences in the histological types and disease stages in cancer patients evaluated in these studies; moreover, it highlights a huge metabolic heterogeneity in ovarian cancer.

One of the metabolites that exhibits the highest fold change between HGSOC and normal ovarian tissue in our work is NAA, a compound synthetized from L-aspartate and acetyl-CoA by the enzyme N-Acetyltransferase 8 Like (NAT8L) and degraded into L-aspartate and acetate by aspartoacylase (ASPA). Although NAA was previously thought to be specifically involved in brain metabolism, a high concentration of this compound was detected in multiple types of cancers, including ovarian cancer [59]. Notably, NAT8L expression was associated with poor survival and increased tumor growth [56]. Fong et al. reported that the NAA levels were significantly higher in the metastatic cancer compared to the primary ovarian cancer [57]. Despite the accumulation of evidence for the high levels of NAA in solid tumors, its biochemical role remains elusive. Menga et al. described an interesting metabolic cross-talk in which NAA is secreted by glutaminolytic ovarian cancer cells to the tumor milieu and enforces the expression of glutamine synthase (catalyzing production of glutamine from glutamate and ammonia) in tumor-associated macrophages [60]. Although in our work the NAA level was found to exhibit a moderate correlation with tumor purity, a high amount of this metabolite was also detected in several samples composed mainly of fibrotic stroma, in agreement with the secretion of this metabolite to the microenvironment. The HR MAS NMR analysis of the tumor samples with high fractions of inflammatory cells could provide important information on the role of NAA in the metabolism of ovarian cancer. Unfortunately, such samples were not available in our study.

Interestingly, NMR analysis of NAT8L overexpressing LLC1 cells revealed a substantial increase in UDP-N-acetylglucosamine (UDP-GlcNac) under glucose starvation conditions [61]. However, it was not possible to link the observed changes in NAA and nucleotide sugars using known metabolic pathways [61]. Uridine nucleotides (contributing to the signals at 5.97, 7.95, and 8.08 ppm) and the associated UDP sugars [contributing to the singlet at 2.07 ppm, to the signal at 5.52 ppm, and to the signals at 5.97, and 7.95 ppm (overlapping with UDP/UTP/UMP nucleotides)] showed significant changes between the cancer samples and the specimens excised from normal ovary and benign lesions in our work. A careful assignment of these signals in ovarian cancer A2780 cells using spiking experiments revealed that UDP-GlcNac and UDP-GalNAc (UDP-N-acetylgalactosamine) contribute to the singlet at 2.07 ppm and to the integrated area of 5.50 to 5.57 ppm [62]. The observed changes correspond well to the known aberrations of nucleotide metabolism in cancer [63] and could be related to the increased flux through the hexosamine biosynthetic pathway [64]. UDP-GlcNAc and UDP-GalNAc are the end products of this pathway used for protein O-GlcNAcylation—a post-trantional reversible modification involving the binding of N-acetylglucosamine to serine or threonine residues associated with cancer progression and metastasis. The addition is catalyzed by O-GlcNAc transferase (OGT), while the removal is catalyzed by O-GlcNAcase (OGA). Thus, the changes in UDP sugars could also be related to the aberrant levels of these enzymes. de Queiroz et al. found downregulation of O-GlcNAcylated proteins in ovarian tumors compared to normal tissue [65], while the decreased OGT and O-GlcNAc levels were found in the tissue samples excised from chemo-resistant patients compared to the sensitive ones [66]. On the other hand, OGT silencing inhibited the migration of the cultured SKOV3 cells (originally reported to be derived from the ascitic fluid of ovarian cancer [67] and classified as “unlikely HGSOC” [68]), and 59M cells (derived from the ascitic fluid of endometrioid carcinoma of ovary, with clear cell components [69]) [70]. Another enzyme involved in the nucleotide sugar metabolism, UDP-glucose dehydrogenase (UGDH), which catalyzes the oxidation of UDP-glucose to UDP-glucuronate, was shown to be upregulated in highly metastatic ovarian cancer TOV21G cells (established from stage 3 clear cell ovarian carcinoma [71]) and in tissue samples excised from clear cell carcinoma and mucinous ovarian carcinoma [72]. Interestingly, the survival of cancer cells overexpressing UGDH was shown to be critically dependent on the UDP-glucuronate decarboxylase 1 (UXS1) enzyme that converts toxic UDP-glucuronate to UDP-xylose [73]. According to the ‘kitchen sink’ model, disruption of UXS1 is expected to affect cancer but not normal cells [73]. The UDP-glucuronate was shown to contribute to the signal at 5.61 ppm in the CPMG spectra [62]. The multivariate analysis performed in our work reveals an increased intensity in this spectral region in the cancer samples compared to the non-transformed ones. However, this result should be interpreted with caution due to the very low signal-to-noise in this spectral area. Although it is now accepted that HGSOC originates from the fallopian tubes rather than from ovarian surface epithelial cells [74], comparison of tumor samples with various unaffected tissues (including normal ovarian tissue) is of great importance in identifying tumor-specific therapeutic targets [73].

Dória et al. and Sans et al. reported on the important role of the phospholipid biosynthesis pathway (involving phosphatidic acid and phosphatidylethanonolamine) in the differentiation between ovarian cancer and normal samples (excised from the ovary, fallopian tube, and peritoneum) and in the delineation of epithelial and stromal regions within ovarian tumors [35,36]. Although in our work a multivariate analysis showed that phosphocholine was slightly increased in cancer relative to normal ovary, the univariate analysis did not confirm this change. On the contrary, Garg et al. found a lower level of this metabolite in HGSOC than in normal tissue [45]. Our study shows that phospholipids are the most important metabolites in the distinction between the samples characterized by the high tumor purity and the samples composed mainly of fibrotic stroma. Furthermore, the regression analysis revealed a strong correlation between phosphocholine level and cancer cell fraction. The ‘cholinic phenotype’ (apparent as increased phosphocholine and the total amount of choline-containing compounds) was shown to be related to the activation of choline kinase α (involved in a biosynthetic Kennedy pathway) and phosphatidylcholine-specific phospholipase C (participating in the phosphatidylcholine degradation pathway) in ovarian cancer [75]. Interestingly, the silencing of choline kinase α impaired the growth of ovarian cancer cells and was associated with decreased glutathione level, a compound that participates in antioxidant defense mechanisms and which plays an important role in chemo-restistance [76]. Along with an increased level of choline-containing compounds, we also obtained an elevation of the phosphoethanolamine signal (with a possible contribution from serine) in the samples characterized by a high cancer purity. The accumulation of this metabolite was associated with an increased expression of ethanolamine kinase 2 (ETNK2) that converts ethanolamine to phosphoethanolamine in pancreatic and breast cancer cells [77].

The higher level of most of the compounds evaluated in the samples representative of cancer (cancer purity > 60%) compared to those representative of the fibrotic stroma obtained by us from multivariate analysis could be associated with the increased metabolism rate in the former samples. However, apart from the mentioned changes in the choline-containing compounds and phosphooethanolamine, the univariate analysis confirmed only the changes in uridine-derived nucleotides and/or associated UDP sugars, threonine, and glutamine. Importantly, the Pearson correlation coefficient between the levels of these metabolites and the percentage of cancer in the samples (containing mainly cancer and fibrotic stroma) exceeds 0.6. In addition, the upward lactate and glycine trends in the samples characterized by a high tumor purity were detected. Interestingly, SHMT1 knockdown resulted in an unexpected reduction in the metabolites involved in the nucleotide sugar metabolic route (including UDP-GlcNAc) in ovarian cancer cells in a study conducted by Gupta et al., suggesting a possible link between this pathway and serine/glycine metabolism [54].

Our study shows that the fibrotic stroma within the HGSOC lesions could be differentiated from the fibrotic stroma in the benign lesions. The multivariate and univariate analyses of the CPMG spectra showed higher levels of glutamine, glutamate, acetate, lysine, and alanine in the former group of samples. The corresponding analyses of the J-resolved spectra revealed elevated leucine and isoleucine in these samples. CAFs, as one of the main cellular components in the tumor microenvironment of malignant tumors, actively participate in the disease progression and metastasis [32,33]. Initial works on the interaction between these cells and cancer cells were concentrated mainly on increased autophagy in CAFs and the ‘reverse Warburg effect’ that involves the secretion of lactate, the ketone bodies, and amino acids by stromal cells and the use of these compounds for the metabolic demands of cancer cells [78]. The increase in the lactate amount in the fibrotic stroma in HGSOC tumors (identified by the multivariate model, but not confirmed as statistically significant in the univariate analysis) could be interpreted as one of the traits of this process. However, accumulating evidence indicates that CAFs cooperate with cancer cells by exploiting a variety of mechanisms. Yang et. al. found the higher expressions of glutamine synthetase and several amino acid transferases (glutamic-oxaloacetic transaminase 1/2, GOT ½; branch chain amino acid transaminase 1, BCAT1) in the micro-dissected fibroblastic stroma of HGSOC tumors compared to normal ovarian fibroblasts [79]. Moreover, CAFs were characterized by the increased expression of genes encoding glycolysis, TCA, and the electron transport chain. Yang et al. identified an interesting interplay between CAFs and cancer cells, whereby CAF-synthetized glutamine was secreted into the tumor microenvironment, imported to cancer cells, and converted to glutamate (via glutaminase) for TCA cycle anaplerosis [79]. Glutamate produced in cancer cells was also shown to be secreted into the tumor microenvironment and used by CAFs for glutamine synthesis. The observed by us increased glutamate and glutamine in the desmoplastic stroma in malignant tumors compared to the stroma in the benign ones could be related to the described cross-talk. The disruption of this metabolic cycle by simultaneous targeting glutamine synthase in CAFs and glutaminase in cancer cells was shown to reduce the tumor growth in an ovarian cancer mouse model [79]. Yang et al. also found that BCAA were among the major nitrogen donors for glutamine synthesis in CAFs [79]. An important role for the BCAA metabolism was also highlighted in fibroblasts associated with pancreatic cancer [80], while alanine secreted by the stroma-associated pancreatic stellate cancer was shown to act as an alternative carbon source in the metabolism of pancreatic cancer cells [81]. Interestingly, several metabolites found in our work to be increased in desmoplastic stroma in HGSOC tumors relative to the fibrotic stroma in the benign tumors (alanine, glutamine, glutamate, acetate, leucine, isoleucine) were detected in the metabolic cargo carried by exosomes derived from patients with prostate and pancreatic cancer [82].

Although our analysis revealed that the variability in tumor purity affects HR MAS NMR spectral profiles, the inter-patient variation was higher than the intra-tumoral one, despite the similar histological composition of the samples excised from different patients. This strengthens the role of metabolomics as a technique that provides information beyond standard histopathology, which could be correlated with the clinical history of the patients (drug response and overall survival) in the search for predictive biomarkers. Our findings generally agree with the results obtained from the analysis of intra-tumoral and inter-patient metabolic variability in breast cancer [29,30,31]. However, Park et al. reported that the levels of phosphocholine and phosphoethanolamine, the compounds found by us to be strongly associated with tumor purity, were not concordant between the center of the tumor and the periphery [31]. The metabolic properties of the stromal and epithelial compartments within HGSOC tumors were also shown to be location-dependent (periphery vs. tumor center) and related to tumor stiffness [83]. In our preliminary study, the tissue samples were excised mainly from the tumor periphery, while the analysis of intra-tumoral heterogeneity was performed based on the samples obtained from the neighboring regions within the tumors. In the near future, we plan to modify the sample collection protocol to obtain information on the variability in metabolic profiles of the samples excised at distal locations within the malignant and benign tumors at known distances from the tumor centers. The expansion of the current database is necessary to overcome one of the main limitations of our study—the small number of analyzed samples.

In conclusion, this is the first study merging HR MAS NMR metabolic profiling and standard histopathology in the analysis of the metabolic properties of HGSOC, benign tumors, and normal ovary samples. The multivariate and univariate analyses showed that the metabolic profile of HGSOC differs from that of the non-transformed tissue. The HR MAS NMR spectra of the samples excised from malignant tumors depend on the tumor purity. The most important features that distinguish the samples with a high percentage of cancer cells (greater than 60%) from the samples containing mainly desmoplastic stroma are the increased levels of phosphoethanolamine/serine, phosphocholine/glycerophosphocholine, threonine, UDP/UTP/UMP nucleotides, and/or UDP nucleotide sugars. Desmoplastic stroma within the HGSOC lesions could be differentiated from the fibrotic stroma in the benign lesions based on the higher levels of glutamine, glutamate, acetate, lysine, alanine, leucine, and isoleucine in the former group of samples. The inter-patient variation was observed to be higher than the intra-tumoral one despite the similar histological composition of the samples excised from different patients.

## 4. Materials and Methods

### 4.1. Collection of Tissue Samples

The study was approved by the Bioethics Committee of the Medical University of Silesia in Katowice (protocol code PCN/0/22/KB1/83/I/20/21) and informed consent was obtained from all patients. All research was carried out in accordance with the relevant guidelines and regulations.

The HGSOC tumor tissue samples were harvested during surgical procedures (surgical staging/debulking surgery), while the benign tumor samples were collected during salpingo-oophorectomy or hysterectomy with salpingo-oophorectomy due to non-malignant tumors. The patients qualified to the control group donated normal ovarian tissue samples excised during salpingo-oophorectomy or hysterectomy with salpingo-oophorectomy due to non-malignant indications, such as uterine leiomyomas and genital prolapse (no neoplastic changes as a result of histopathological examination).

The tissue samples were frozen (−80 °C) until NMR analysis.

### 4.2. HR MAS NMR

The HR-MAS ^1^H NMR experiments were performed on a Bruker Avance III 400 MHz spectrometer (Bruker BioSpin GmbH, Karlsruhe, Germany) equipped with a ^1^H optimized 4-mm ^1^H/^13^C MAS probe. The frozen tissue samples were cut, weighted, and placed in 30 μL disposable Kel-F inserts filled with a 5 μL cold solution of 25 mM sodium formate in D_2_0 for shimming and locking procedures. The inserts were introduced into 4 mm zirconium HR-MAS rotors. The measurement protocol included the acquisition of the following:

- The Carr–Purcell–Meiboom–Gill (CPMG) spectra measured using a cpmgpr1d sequence (a relaxation delay of 4 s, an effective echo time of 90 ms, a spectral width of 20 ppm, 65 k points, an acquisition time of 4.08 s, and 256 scans).
- The two-dimensional ^1^H-^1^H J-resolved spectra using a jresgpprqf sequence (8k time domain data points, 40 increments, 8 scans per increment, a spectral width of 20 ppm (F2) and 78.2 Hz (F1), and a relaxation delay of 2s).

The spectra were acquired at 4 °C (calibrated with methanol) with magic-angle spinning at 4000 Hz. After HR-MAS NMR measurements, the samples were frozen for histopathological evaluation of the tissue content.

### 4.3. Histopathological Verification

Preparation of the post-^1^H HR MAS NMR samples for histopathological examination included fixing in 10% formalin and embedding in paraffin. The samples were cut into multiple 3 µm-thick cross-sections and stained with hematoxylin and eosin (H&E). The percentage fractions of cancer cells, fibrosis, necrosis, inflammation, calcification, vessels, fatty tissue, and normal ovary tissue were evaluated by an experienced pathologist.

### 4.4. Data Preprocessing

The preprocessing of the NMR data was performed in Topspin 3.1 software (Bruker BioSpin, Rheinstetten, Germany). Raw HR MAS NMR CPMG spectra were apodized with an exponential function [line broadening of 0.6 Hz was applied for the aliphatic region (0.8–4.8 ppm), while a line broadening of 3 Hz for the aromatic region (5.2–8.4 ppm)], Fourier-transformed, phased, and baseline corrected. The 2D J-resolved spectra were apodized with SEM function, Fourier transformed, tilted by 45°, baseline corrected, and symmetrized. The CPMG spectra and one-dimensional projections of 2D J-resolved spectra onto the chemical shift axis (p-Jres) were referenced to the formate peak (at 8.44 ppm) in the MestReNova 10 software (Mestrelab Research, Santiago, Spain). Then, fine peak alignment was performed using the FFT/peak matching method in SpecAlign 2.4 software (Oxford University, Oxford, UK) [84]. The spectra were normalized using the sample weight.

The processed spectra were Pareto scaled and subjected to a multivariate analysis in SIMCA 17.0 software (Sartorius Stedim Biotech, Umea, Sweden).

### 4.5. Multivariate Analysis

Principal component analysis (PCA) was used as an unsupervised technique, while orthogonal partial least squares discriminant analysis (OPLS-DA) and orthogonal partial least squares regression (OPLSR) were used as supervised techniques. The results are shown in the score and loading plots of the obtained models. The multivariate models were described by the fraction of the explained variation (R^2^) and the fraction of the variation predicted by seven-fold cross validation (Q^2^). The statistical significance of the created models was tested using cross validation analysis of variance (CV-ANOVA).

### 4.6. Univariate Analysis

The metabolic differences between different tissue components (cancer, fibrotic stroma within the malignant tumors, fibrotic stroma within the benign tumors, and normal ovarian tissue) were also analyzed using univariate statistics. The signal integrals corresponding to specific metabolites were subjected to the Kruskal–Wallis test followed by multiple comparisons of the mean ranks. *p* values of <0.05 were accepted as statistically significant, while those in the range from 0.05 to 0.1 were considered to indicate trends. The relationship between the metabolic levels (in terms of the spectral integrals) and tumor purity in the samples included in a given OPLSR model was analyzed using a linear regression.

The signal integrals corresponding to the metabolite levels were determined from the CPMG and p-Jres spectra using a direct integration technique in the MestReNova software. Additionally, an automatic signal deconvolution was performed in the region of 3.15 to 3.25 ppm in the CPMG spectra for a more precise estimation of the integrals of the overlapping phosphocholine and glycerophosphocholine signals.

The results are interpreted using the major metabolites that contribute to the spectral integrals identified based on the 2D HR-MAS NMR spectra (^1^H-^13^C HSQC, ^1^H-^1^H TOCSY) measured for selected samples, publicly available databases [85,86], the reference compounds library in Chenomx NMR Suite 9.0 Professional software (Chenomx Inc., Edmonton, AB, Canada), and the published data [22,62]. However, the possible contributions from less abundant metabolites to the integrated areas cannot be excluded. A considerable overlap of some metabolites is marked with ‘slash’ (for example: phosphocholine/glycerophosphocholine).

### 4.7. Joint Univariate and Multivariate Analysis

The metabolites detected in both the CPMG and p-Jres spectra were considered to discriminate between the classes if they were assigned p(corr)[1] values above 0.5 in OPLS-DA models computed based on both types of spectra and were found to be significantly changed in the univariate analysis of the integrals determined from at least one type of spectra. The compounds detected in the either CPMG or p-Jres spectra were considered important for the discrimination of the groups if they were assigned p(corr)[1] values greater than 0.5 in the appropriate OPLS-DA models and if the statistical significance of the change was also confirmed in the univariate analysis.

## Figures and Tables

**Table 1:** The clinical–pathological characteristics of the studied groups.

Group	BMI [kg/m^2^] Median (I–III Quartile)	Age [years] Median (I–III Quartile)	FIGO Stage —Number of Patients	Menopausal Status
HGSOC	28.13 (24.91–30.28)	58.5 (49–68)	I—1 II—4 III—20 IV—19	Pre-menopausal: 12/44 (27%) Post-menopausal: 32/44 (73%)
Benign ovarian tumors *	28.86 (26.77–35.60)	59.5 (48–72)	-	Pre-menopausal: 6/18 (33%) Post-menopausal: 12/18 (67%)
Control group	29.72 (28.73–31.61)	48 (47–62)	-	Pre-menopausal: 5/13 (38%) Post-menopausal: 8/13 (62%)

* Endometrioid cystadenoma ovary (*n* = 2), corpus luteum cyst (*n* = 1), mature teratoma (*n* = 2), simple ovary cyst (*n* = 1), ovarian fibroma (*n* = 1), fibrothecoma ovary (*n* = 1), serous adenofibroma ovary (*n* = 2), serous cystadenoma ovary (*n* = 3), mucinous cystadenoma ovary (*n* = 1), serous cystadenofibroma ovary (*n* = 4). HGSOC—high-grade serous ovarian cancer, BMI—body mass index, FIGO—the International Federation of Gynecology and Obstetrics.

**Table 2:** Results of the histological examinations of the samples after the HR MAS NMR measurements.

Samples		Number of Samples/Number of Patients	Statistics	Cancer Cells [%]	Epithelial Component in the Benign Tumors [%]	Necrosis [%]	Inflammation [%]	Fibrosis [%]	Calcifications [%]	Normal Ovary [%]	Vessels [%]	Fatty Tissue [%]
HGSOC	C 0%	17/11	Median	0	0	0	0	5	0	75	0	0
			Minimum	0	0	0	0	0	0	0	0	0
			Maximum	0	0	100	15	85	5	100	30	20
			I quartile	0	0	0	0	0	0	0	0	0
			III quartile	0	0	0	10	20	0	95	10	0
	C 1–19%	22/20	Median	5	0	0	0	0	0	50	0	0
			Minimum	2	0	0	0	0	0	0	0	0
			Maximum	15	0	95	15	90	5	98	30	10
			I quartile	5	0	0	0	0	0	0	0	0
			III quartile	10	0	0	5	80	0	90	5	0
	C 20–100%	55/23	Median	40	0	0	0	45	0	0	0	0
			Minimum	20	0	0	0	0	0	0	0	0
			Maximum	80	0	25	15	80	10	50	15	10
			I quartile	30	0	0	0	25	0	0	0	0
			III quartile	60	0	5	10	60	0	0	0	0
Control group		13/13	Median	0	0	0	0	0	0	100	0	0
			Minimum	0	0	0	0	0	0	90	0	0
			Maximum	0	0	0	5	0	0	100	10	0
			I quartile	0	0	0	0	0	0	100	0	0
			III quartile	0	0	0	0	0	0	100	0	0
Benign tumors *		11/7	Median	0	5	0	0	90	0	0	0	0
			Minimum	0	0	0	0	80	0	0	0	0
			Maximum	0	10	0	5	100	0	10	5	0
			I quartile	0	5	0	0	90	0	0	0	0
			III quartile	0	10	0	5	95	0	0	0	0

* Samples excised from 6 patients with benign serous tumors and 1 fibroma. HGSOC—high-grade serous ovarian cancer, C 0%—the samples containing no cancer cells, C 1–19%—the samples characterized by a cancer content of 1–19%, C 20–100%—the samples characterized by a cancer content of 20–100%.

**Table 3:** Histological composition of the samples that are representative of particular tissue components.

Tissue Component	Criteria
Cancer (HGSOC) compartment	Fraction of cancer cells ≥ 60%, normal ovary fraction = 0%
Fibrotic stroma within malignant (HGSOC) tumors	Cancer cell fraction ≤ 10%, Fibrosis fraction ≥ 70%, Normal ovary fraction = 0%
Fibrotic stroma within benign tumors	Epithelial compartment fraction ≤ 10%, fibrosis fraction ≥ 80%, Normal ovary fraction ≤ 10%,
Normal ovary (control group)	Normal ovary tissue fraction ≥ 90%
Normal ovary (cancer patients)	Normal ovary tissue fraction ≥ 90%
Necrosis	Necrosis fraction (≥ 90%)
Non-tumoral fibrous connective tissue (benign non-neoplastic lesion patients)	Fibrosis fraction ≥ 75% Normal ovary fraction = 0%

**Table 4:** The numbers of the samples that are representative of individual tissue components and those included in PCA model 5a.

Tissue Component	Number of the Samples Fulfilling the Criteria Presented in Table 3/ Number of Patients	Number of the Samples in Included in the Model PCA 5a
Cancer (HGSOC) compartment	18/10	10
Fibrotic stroma within malignant (HGSOC) tumors	11/11	11
Fibrotic stroma within benign tumors	11/7	8
Normal ovary (control group)	13/13	13
Normal ovary (cancer patients)	13/7	7
Necrosis	4/2	-
Non-tumoral fibrous tissue (benign non-neoplastic lesions patients)	6/6	6

**Table 5:** The OPLS-DA models built to distinguish between various tissue components.

Tissue Components Being Differentiated in a Given Model	Spectra	Model Name
Cancer and normal ovarian tissues excised from the control group	CPMG	OPLS-DA 1_CPMG_
	J-res	OPLS-DA 1_J-res_
Cancer and fibrotic stroma in the malignant tumors	CPMG	OPLS-DA 3_CPMG_
	J-res	OPLS-DA 3_J-res_
Fibrotic stroma in the malignant tumors and fibrotic stroma in the benign tumors	CPMG	OPLS-DA 4_CPMG_
	J-res	OPLS-DA 4_J-res_
Normal ovarian tissue and fibrosis in the benign tumors	CPMG	OPLS-DA 5_CPMG_
	J-res	OPLS-DA 5_J-res_
Normal ovarian tissue and fibrosis within the malignant lesions	CPMG	OPLS-DA 6_CPMG_
	J-res	OPLS-DA 6_J-res_

CPMG—Carr–Purcell–Meiboom–Gill, J-res—J-resolved, OPLS-DA—orthogonal partial least squares discriminant analysis.

**Table 6:** The results of linear regression analysis between the metabolite levels determined using HR MAS CPMG NMR spectra and a cancer cell fraction.

Metabolite (Chemical Shift)	Pearson Correlation Coefficient r, *p* Value	Integral Region [ppm]
Glutathione (4.57 ppm)	r = 0.47; *p* = 0.0171	4.539–4.602
Threonine (4.25 ppm)	r = 0.47; *p* = 0.0158	4.20–4.28
Lactate (4.12 ppm)	r = 0.59; *p* = 0.0021	4.074–4.160
Myo-inositol (4.05 ppm)	r = 0.46; *p* = 0.0211	4.025–4.075
Phosphoethanolamine/Serine (3.97 ppm)	r = 0.68; *p* = 0.0002	3.934–4.005
Glycine (3.54 ppm)	r = 0.49; *p* = 0.0123	3.544–3.560
Glycerophosphocholine (3.22 ppm)	r = 0.54; *p* = 0.0056 *	According to automatic deconvolution algorithm
Phosphocholine (3.21 ppm)	r = 0.71; *p* = 0.00007 *	According to automatic deconvolution algorithm
Phosphocholine (3.21 ppm) + Glycerophosphocholine (3.22 ppm)	r = 0.82; *p* = 0.00000 *	According to automatic deconvolution algorithm
Ethanolamine (3.12 ppm)	r = 0.43; *p* = 0.0304	3.098–3.151
Creatine (3.02 ppm)	r = 0.44; *p* = 0.0268	2.998–3.048
Glutamine (2.44 ppm)	r = 0.66; *p* = 0.0003	2.407–2.472
Succinate (2.4 ppm)	r = 0.44; *p* = 0.0295	2.3878–2.410
Uridine nucleotides (UMP/UDP/UTP) and/or nucleotide UDP sugars (7.96 ppm)	r = 0.67; *p* = 0.0002	7.916–8.002
Uridine nucleotides (UMP/UDP/UTP) and/or nucleotide UDP sugars (5.97 ppm)	r = 0.69; *p* = 0.0001	5.924–6.013
UMP (8.07 ppm)	r = 0.54; *p* = 0.0055	8.03–8.114

* The spectral integrals obtained using automatic signal deconvolution technique. The abbreviations for metabolites are the same as in the legend of Figure 1.

**Table 7:** The results obtained from joint univariate and multivariate analysis according to the criteria presented in Section 4.7. Additionally, metabolites characterized by VIP values above 1 in both J-res and CPMG models are bolded.

Differentiated Tissue Components	Metabolic Changes
Cancer in reference to normal ovary	Increased **Ala**, **Glu**, **Gln**, **Cre**, **Gly**, **Lac**, Thre *, **Ile**, **Leu**, **Val**, HX, **NAA**, uridine nucleotides and associated nucleotide sugars **, **hTau**, and **Eth**
Cancer in reference to fibrotic stroma within benign tumors	Increased **Ala**, **Glu**, **Gln**, **Cre**, **Gly Lac**, Thre *, **Ile**, Leu, **Val**, HX, **NAA**, uridine nucleotides and associated nucleotide sugars ***, **hTau**, **Lys**, **PE/Ser**, Tyr, Phe, GSH, and **Ace**
Cancer in reference to fibrotic stroma within malignant tumors	Increased **PE/Ser**, **PCho/GPCho**, Thre *, and uridine nucleotides and/or associated nucleotide sugars
Fibrotic stroma within malignant tumors in reference to fibrotic stroma within benign tumors	Increased **Ala**, **Lys**, **Ace**, **Glu**, **Gln**, **Ile**, and **Leu**
Relation between cancer cell fraction and metabolic profile	The higher the cancer cell fraction, the higher the **Lac**, **Gln**, **hTau**, Eth *, **PCho**, **GPCho**, **MI**, **PE/Ser**, uridine nucleotides and/or associated nucleotide sugars ***, **NAA**, and GSH

* VIP > 1 in the OPLS-DA 1_CPMG_ model; ** VIP > 1 in the OPLS-DA 1_CPMG_ model (5.97 ppm) and in the OPLS-DA 1_J-res_ model (singlet at 2.07 ppm); *** VIP > 1 in the OPLS-DA 1_J-res_ model (singlet at 2.07 ppm). The abbreviations for metabolites are the same as in the legend of Figure 1.

## Data Availability

The raw HR MAS NMR data is available in Zenodo: https://zenodo.org/records/13755458 (accessed on 7 October 2024), DOI: 10.5281/zenodo.13755458.

## Supplementary Materials

The following supporting information can be downloaded at: https://www.mdpi.com/article/10.3390/ijms252010903/s1.
