# Supplementary material for: Metabolomic Analysis of Histological Composition Variability of High-Grade Serous Ovarian Cancer Using 1H HR MAS NMR Spectroscopy
Source: Int J Mol Sci. 2024 Oct 10;25(20):10903. doi: 10.3390/ijms252010903 (PMC11507550; doi:10.3390/ijms252010903)
Supplement: Supplementary file 1 [file ijms-25-10903-s001.zip › ijms-3233036-supplementary.pdf]

Supplementary material

Metabolomic Analysis of Histological Composition Variability of High-Grade Serous Ovarian Cancer Using ^{1}H HR MAS NMR Spectroscopy

Agnieszka Skorupa [1,*,†], Mateusz Klimek [2,†], Mateusz Ciszek [1], Sławomir Pakuło [3], Tomasz Cichoń [2], Bartosz Cichoń [2], Łukasz Boguszewicz [1], Andrzej Witek [2] and Maria Sokół [1]

[1] Department of Medical Physics, Maria Skłodowska-Curie National Research Institute of Oncology, Gliwice Branch, 44-102 Gliwice, Poland; mateusz.ciszek@gliwice.nio.gov.pl (M.C.); lukasz.boguszewicz@gliwice.nio.gov.pl (Ł.B.); maria.sokol@gliwice.nio.gov.pl (M.S.);
[2] Department of Gynecology, Obstetrics and Oncological Gynecology, Faculty of Medicine in Katowice, Medical University of Silesia, 40-752 Katowice, Poland; matheaus.klimek@gmail.com (M.K.); cichon.tomasz.ct@gmail.com (T.C.); bartosz.cichon23@gmail.com (B.C.); awitek@sum.edu.pl (A.W.);
[3] Tumor Pathology Department, Maria Skłodowska-Curie National Research Institute of Oncology, Gliwice Branch, 44-102 Gliwice, Poland; slawomir.pakulo@gliwice.nio.gov.pl
* Correspondence: agnieszka.skorupa@gliwice.nio.gov.pl
† These authors contributed equally to this work.

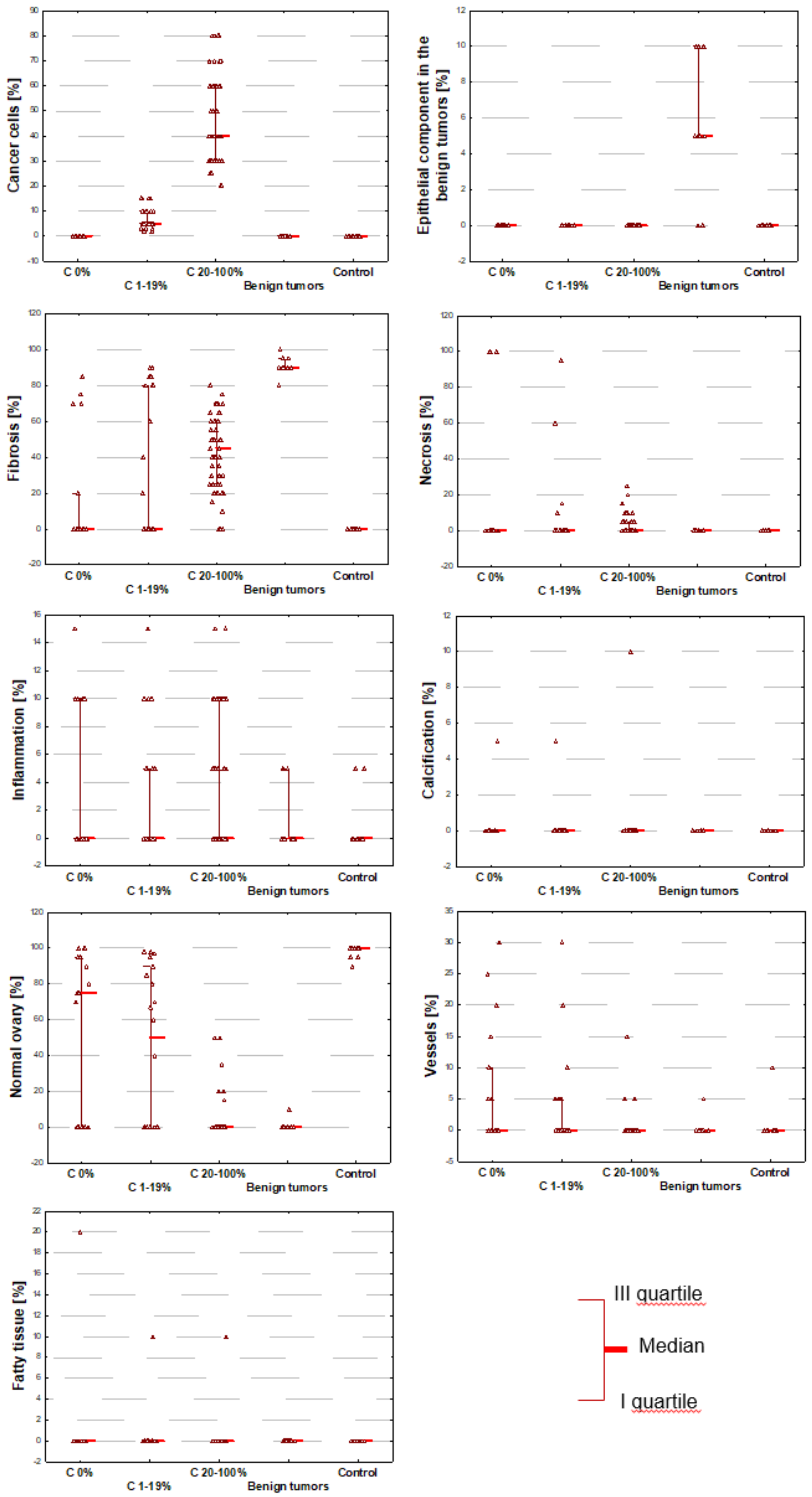

Figure S1. Results of the histological examinations of the samples after the HR MAS NMR measurements, according to Table 2. C 0% - the samples containing no cancer cells, C 1-19% - the samples characterized by a cancer content of 1 - 19%, C 20-100% - the samples characterized by a cancer content of 20 - 100%.

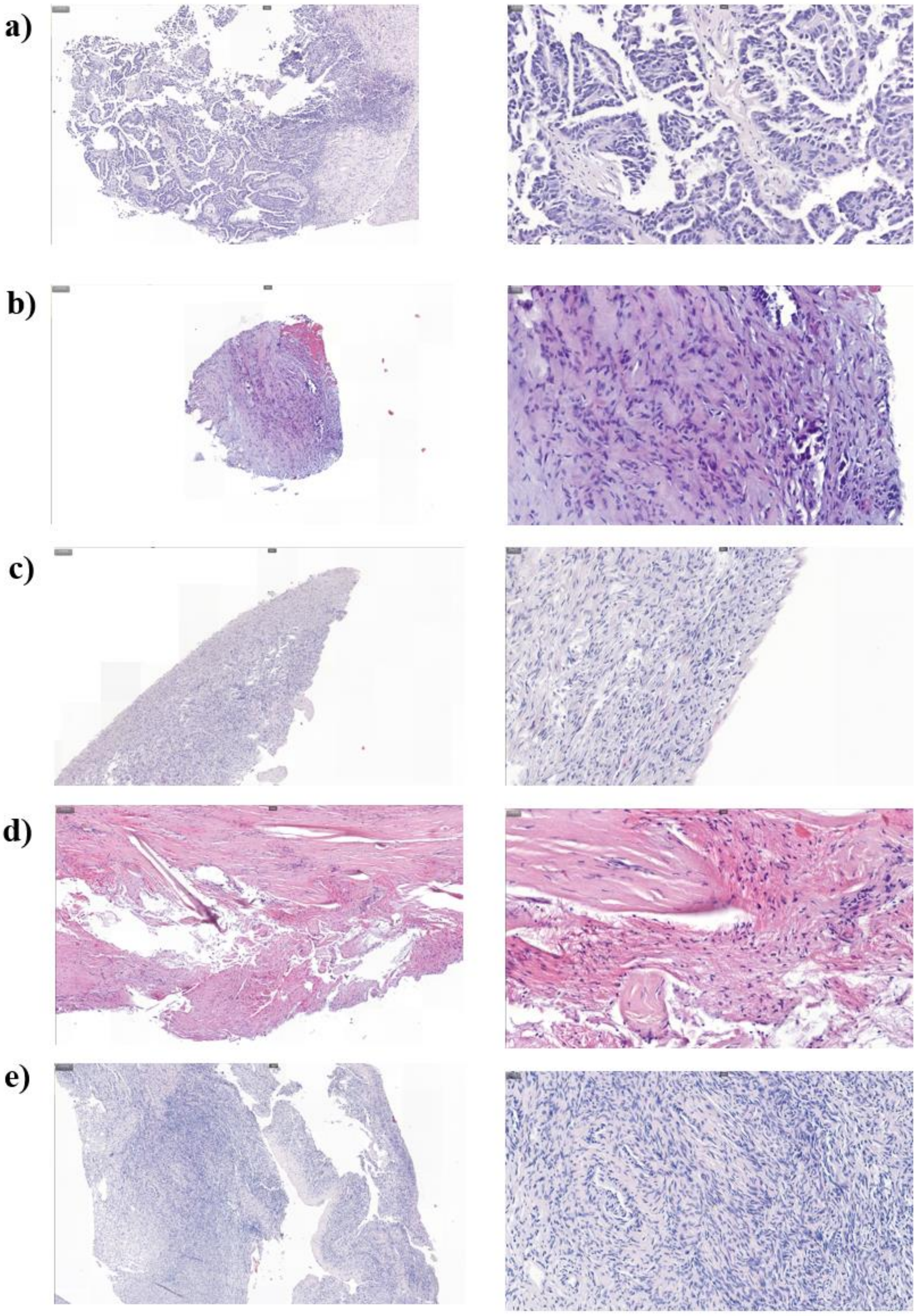

Figure S2. H&E staining of samples representative of (a) HGSOC compartment (magnification: 10x – left, 40x – right), (b) fibrotic stroma within malignant (HGSOC) tumors (magnification: 10x – left, 40x – right), (c) fibrotic stroma within benign tumors (magnification: 10x – left, 40x – right), (d) non-tumoral fibrous connective tissue excised from benign nonneoplastic lesion patients (magnification: 10x – left, 40x – right), (e) normal ovary (magnification: 10x – left, 40x – right).

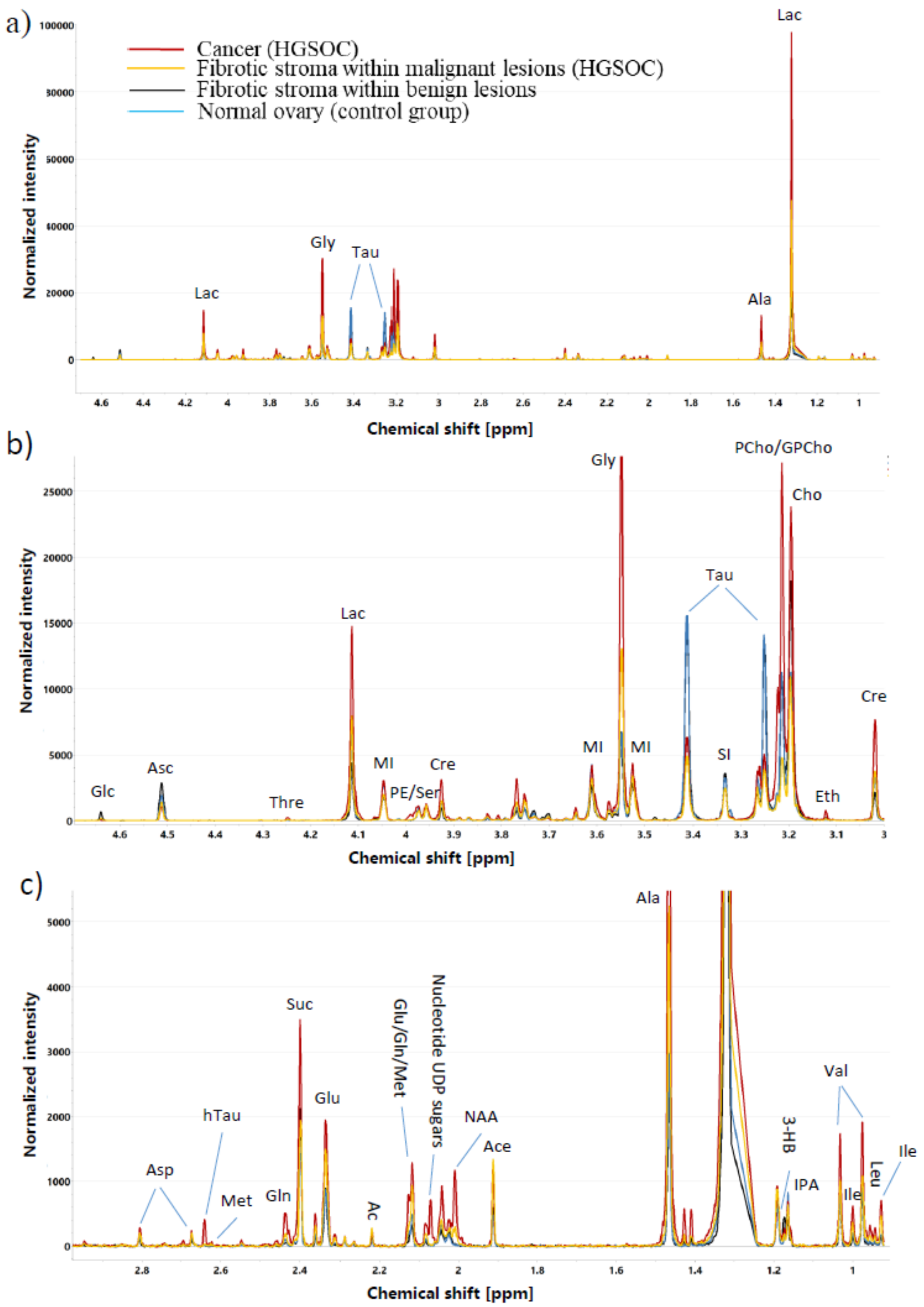

Figure S3. Median p-Jres spectra [regions: 0.7–4.8 ppm (a), 3.0–4.8 ppm (b), 0.7–3.0 ppm (c)] obtained for HGSOC cancer compartment, fibrotic compartment within malignant tumors, fibrotic compartment within benign tumors, normal ovary tissue (samples collected from control group). The abbreviations for metabolites are similar as in the legend of Figure 1.

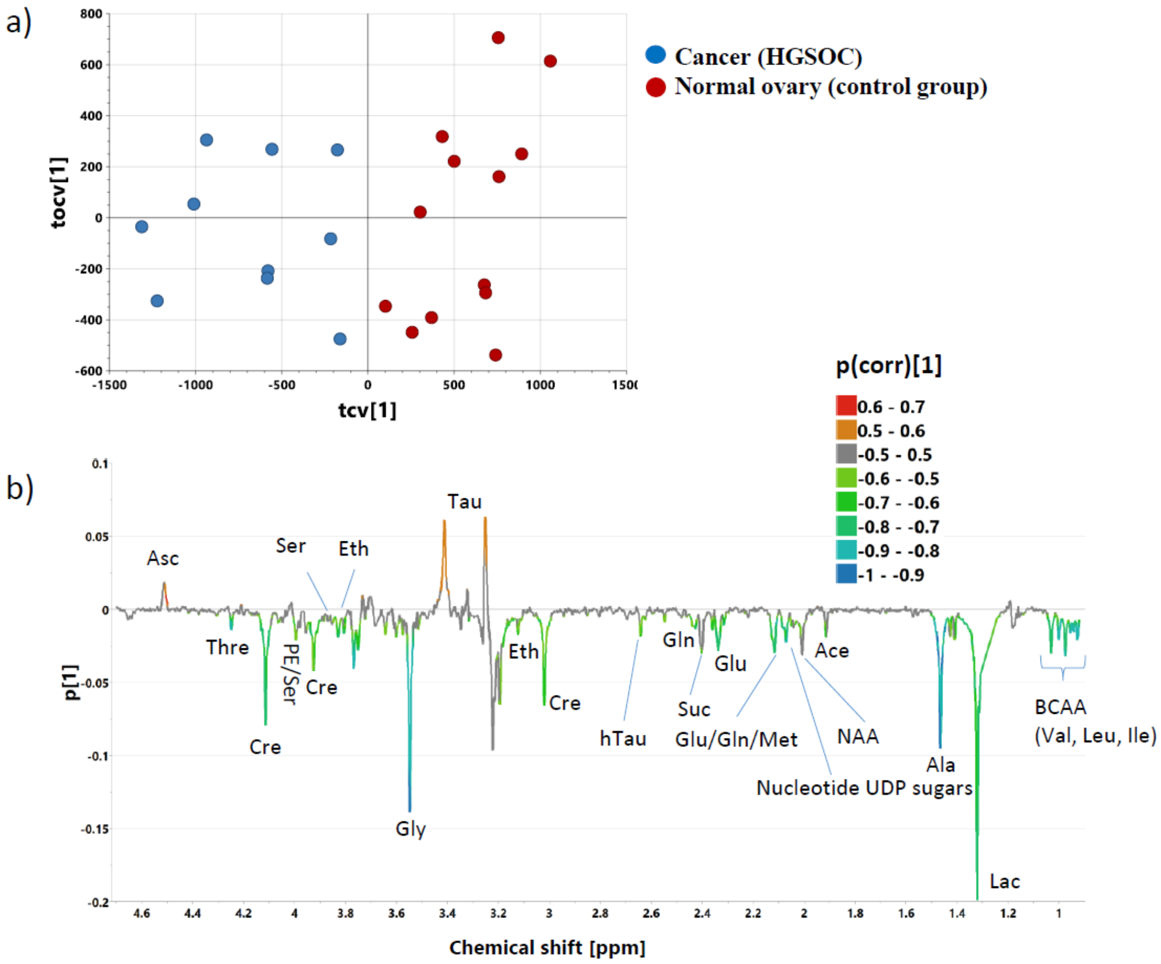

Figure S4. The cross-validated scores (a) and loadings (b) plots obtained from OPLS-DA 1J-res model. The cross-validated scores for the predictive component are denoted as tcv[1], whereas for the orthogonal one - as tocv[1]. The loadings for the predictive component are denoted as p[1]. The signals in the loadings plots are colored according to the p(corr)[1] values (loadings scaled as correlation coefficients between the original data and scores obtained for the first component). The abbreviations for metabolites are similar as in the legend of Figure 1.

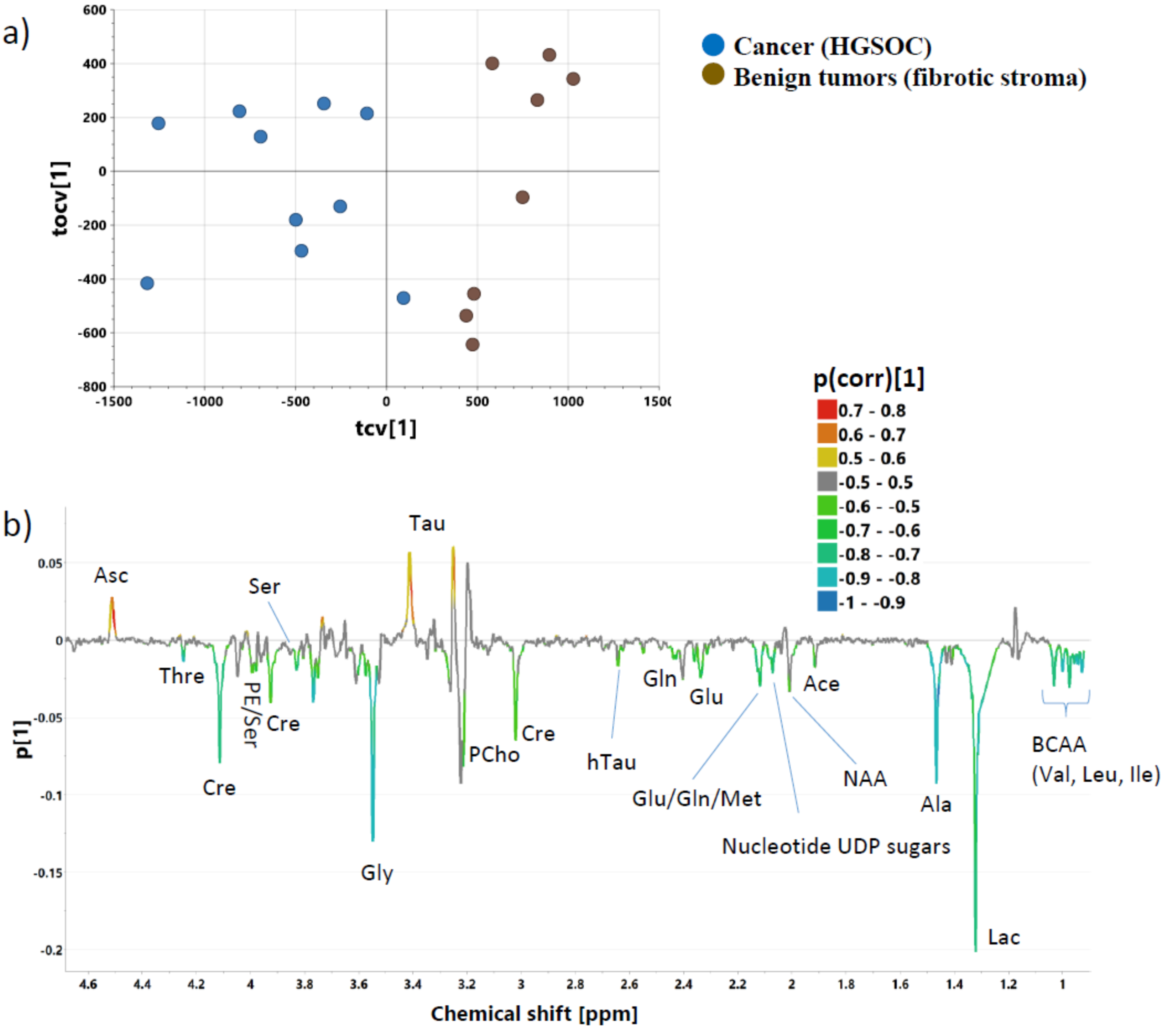

Figure S5. The cross-validated scores (a) and loadings plots (b) obtained from OPLS-DA 2J-res model. The cross-validated scores for the predictive component are denoted as tcv[1], whereas for the orthogonal one - as tocv[1]. The loadings for the predictive component are denoted as p[1]. The signals in the loadings plots are colored according to the p(corr)[1] values (loadings scaled as correlation coefficients between the original data and scores obtained for the first component). The abbreviations for metabolites are similar as in the legend of Figure 1.

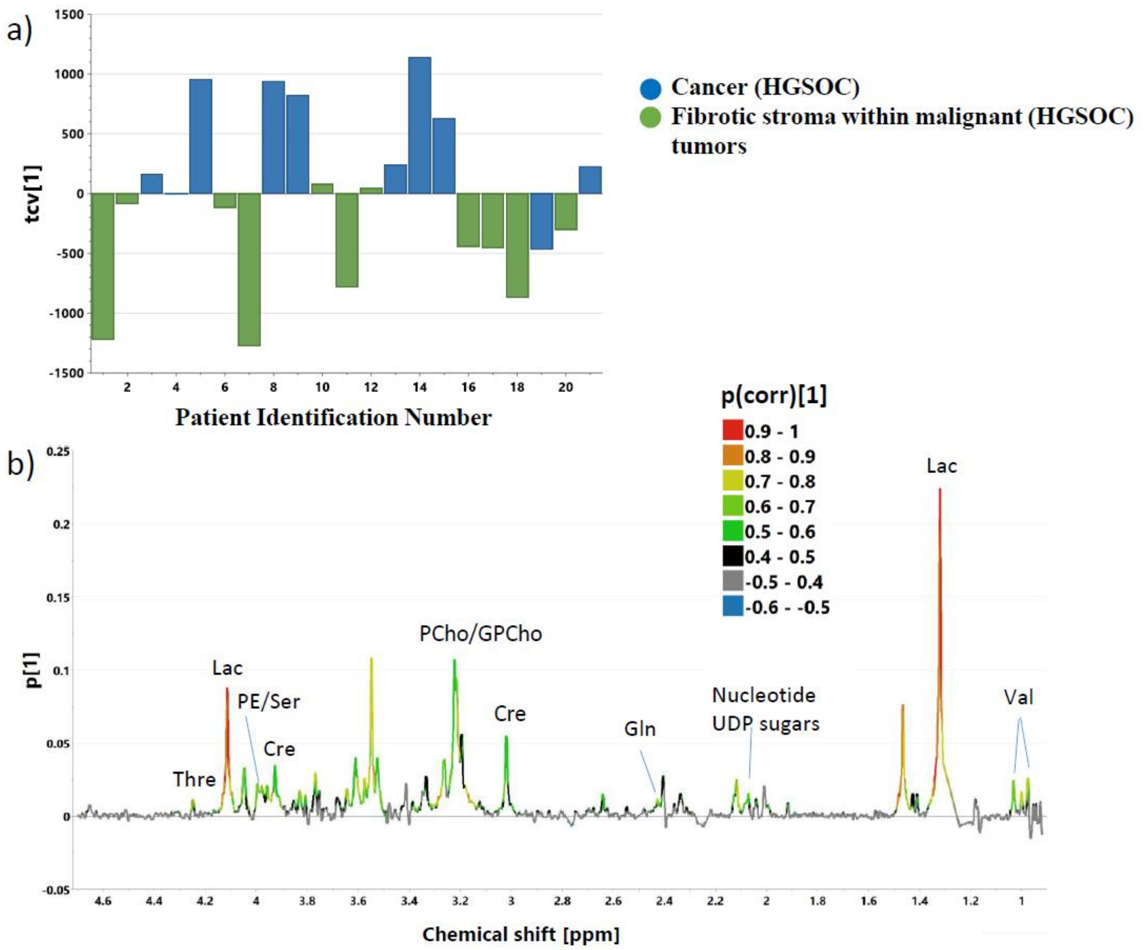

Figure S6. The cross-validated scores (a) and loading plots (b) obtained from OPLS-DA $3_{J\text{-}res}$ model. The cross-validated scores for the predictive component are denoted as tcv[1]. The loadings for the predictive component are denoted as p[1]. The signals in the loadings plots are colored according to the p(corr)[1] values (loadings scaled as correlation coefficients between the original data and scores obtained for the first component). The abbreviations for metabolites similar as in the legend of Figure 1.

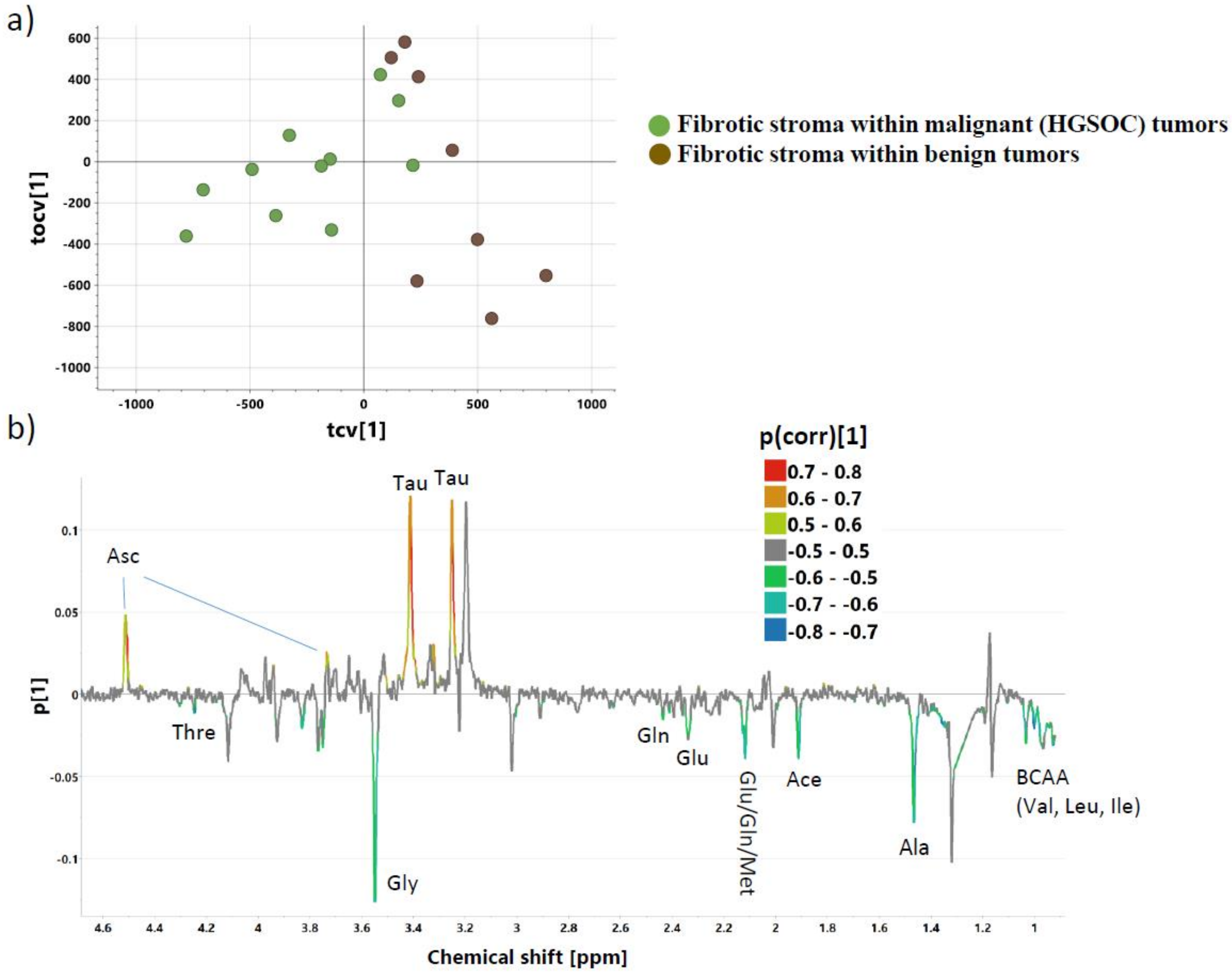

Figure S7. The cross-validated scores (a) and loadings (b) plots obtained from OPLS-DA $4_{J\text{-}Res}$ model. The cross-validated scores for the predictive component are denoted as tcv[1], whereas for the orthogonal one - as tocv[1]. The loadings for the predictive component are denoted as p[1]. The signals in the loadings plots are colored according to the p(corr)[1] values (loadings scaled as correlation coefficients between the original data and scores obtained for the first component). The abbreviations for metabolites are similar as in the legend of Figure 1.

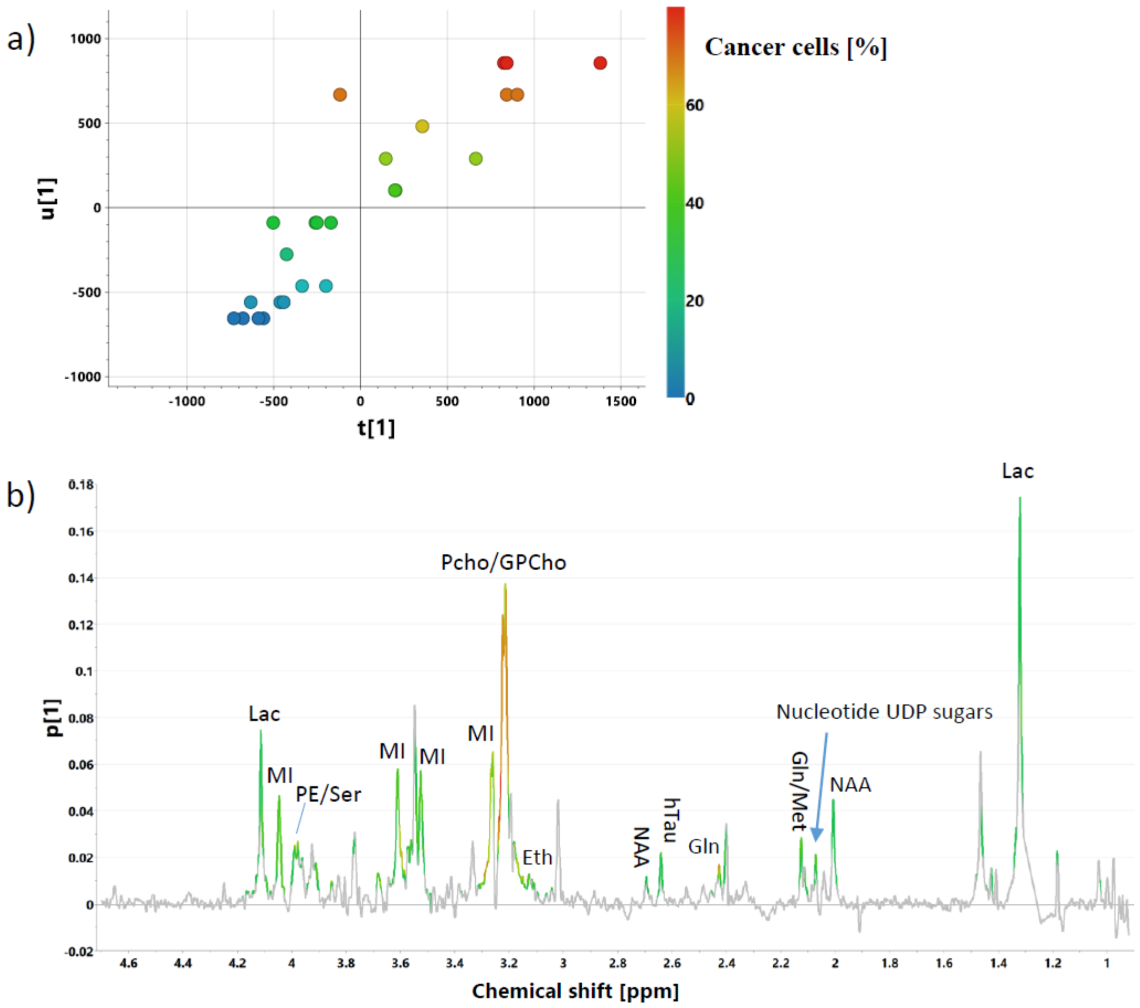

Figure S8. The scores (a) and loadings (b) plots obtained from OPLS$_{J\text{-}res}$ model. The X-scores for the predictive component are denoted as t[1], whereas Y-scores—as u[1].The loadings for the first predictive component are denoted as p[1]. The signals in the loadings plots are colored according to the p(corr)[1] values (loadings scaled as correlation coefficients between the original data and scores obtained for the first component). The abbreviations for metabolites similar as in the legend of Figure 1.

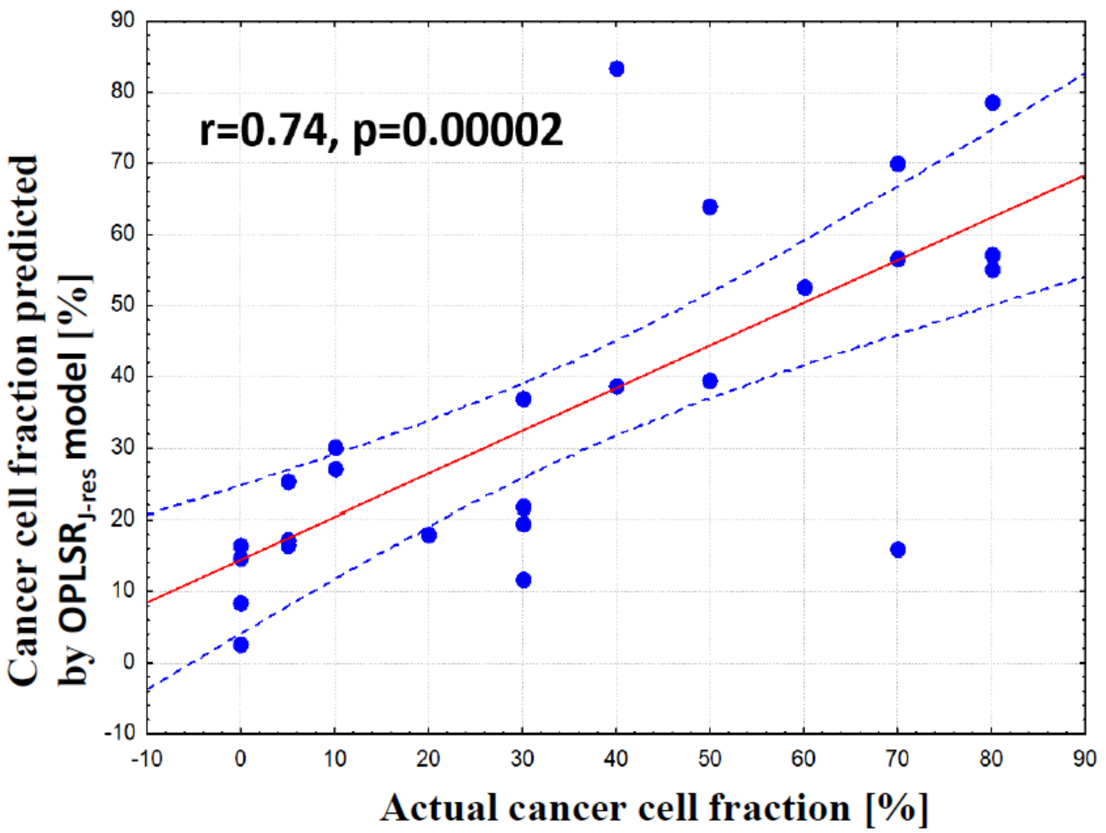

Figure S9. The cancer cells fraction predicted by the OPLSR $_{J\text{-res}}$ model (based on cross-validation) vs. the actual cancer content determined from histological examinations.

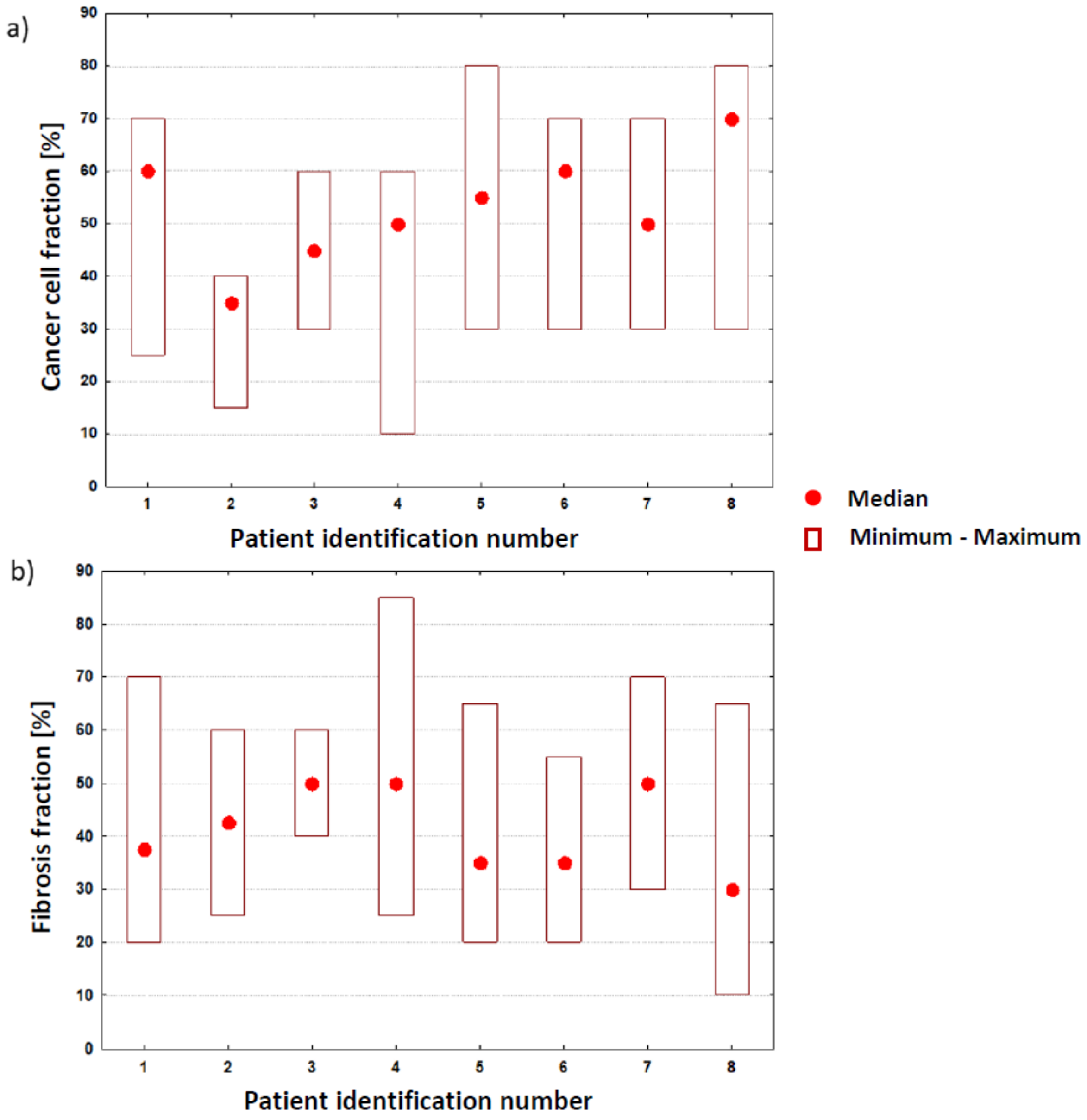

Figure S10. Cancer cell fraction (a) and fibrosis fraction (b) in the samples constituting 'heterogeneity dataset'.

Table S1. Compounds contributing to discrimination between the samples representative of cancer and normal ovary tissue (OPLS-DA 1_{CPMG} model) and between the samples representative of cancer and benign tumors (OPLS-DA 2_{CPMG} model). The fold changes were computed as ratios of the median metabolite levels in the cancerous tissue to the median metabolite level in the normal ovary or in the benign tumors, the p-values were obtained from the multiple comparisons of the mean ranks following the Kruskal-Wallis test.

NS – not significant, ND – not determined, p(corr)[1] - loadings scaled as correlation coefficients between the original data and scores obtained for the first component, VIP - Variable Importance at Projection

Metabolite	Chemical shift [ppm]	HGSOC vs normal ovary				HGSOC vs benign tumors (fibrotic stroma)				Integral region [ppm]
		Model OPLS-DA 1_{CPMG}		Univariate analysis		Model OPLS-DA 2_{CPMG}		Univariate analysis		
		p(corr)[1]	VIP	Fold change	P value	p(corr)[1]	VIP	Fold change	P value	
Alanine	1.46	-0.91	5.91	2.01	0.001606	-0.89	5.26	1.49	0.000035	1.422-1.514
Lysine	1.73	-0.65	1.03	1.15	NS	-0.72	1.09	1.49	0.001153	1.642-1.776
Glutamate	2.34	-0.81	3.16	1.88	0.001721	-0.79	2.88	2.48	0.000227	2.303-2.374
Glutamine	2.44	-0.71	1.71	2.42	0.002521	-0.75	1.75	3.19	0.000014	2.407-2.472
Acetate	1.91	-0.42	1.25	1.56	0.070251	-0.51	1.33	1.69	0.001366	1.90-1.923
2.60–2.70 ppm (Hipotaurine. methione. NAA. asparatate)	2.62	-0.71	1.25	ND	ND	-0.71	1.18	ND	ND	-
	2.64	-0.62	1.32			-0.61	1.22			
	2.68	-0.59	0.94			-0.59	0.94			
	2.70	-0.60	0.79			-0.60	0.76			
Creatine	3.02	-0.66	4.52	1.35	0.010260	-0.64	4.11	2.03	0.000034	2.998-3.048
	3.91	-0.65	2.69	ND	ND	-0.61	2.33	ND	ND	-
Ethanolamine	3.12	-0.72	1.76	1.91	0.003206	-0.58	1.22	1.68	NS	3.098-3.151

Phosphocholine	3.21	-0.41	4.55	1.98*	NS*	-0.66	6.47	3.18*	NS*	According to automatic signal deconvolution
Taurine	3.41	0.56	4.99	0.66	NS	0.51	3.62	0.62	NS	3.376-3.442
Glycine	3.55	-0.89	8.66	2.14	0.000968	-0.86	7.67	3.06	0.000119	3.544-3.560
Phosphoethanolamine/Serine	3.97	-0.23	0.91			-0.58	2.12			
	3.98	-0.42	1.62	1.33	NS	-0.74	3.02	1.88	0.039232	3.934-4.005
	3.99	-0.53	2.32			-0.73	2.92			
Lactate	4.12	-0.75	5.88	1.94	0.027210	-0.78	5.64	2.81	0.000448	4.074-4.160
Threonine	4.25	-0.77	1.34	1.28	0.104526	-0.77	1.31	2.10	0.009542	4.20-4.28
Ascorbate	4.51	0.61	1.67	0.59	0.094034	0.67	1.83	0.57	NS	4.494-4.529
Glutathione	4.57	-0.17	0.23		NS	-0.54	0.72	2.16	0.036279	4.539-4.602
Nucleotide UDP sugars	5.52	-0.74	0.56	9.04	0.015764	-0.74	0.50	3.69	0.031800	5.468-5.562
Nucleotide UDP sugars	5.60	-0.52	0.26	3.10	0.112830	-0.64	0.30	2.67	0.044636	5.564-5.640
Uracil	5.79	-0.59	0.39	1.56	NS	-0.51	0.31	1.40	NS	5.748-5.810
	7.53	-0.61	0.38	1.60	NS	-0.64	0.38	4.51	0.065881	7.470-7.566
UDP/UTP/ UMP/ Nucleotide UDP sugars	5.97	-0.77	1.05	7.92	0.002933	-0.74	0.93	10.70	0.001786	5.924-6.013
UDP/UTP/Nucleotide UDP sugars	7.95	-0.78	0.82	3.18	0.018693	-0.77	0.76	1.69	0.000106	7.916-8.002
UMP	8.08	-0.57	0.47	1.83	0.073284	-0.61	0.47	3.20	0.001486	8.03-8.114
N?	6.11	-0.55	0.51	ND	ND	-0.49	0.42	ND	ND	-
Fumarate	6.51	-0.59	0.38	1.22	NS	-0.55	0.33	1.67	NS	6.479-6.540

Tyrosine	6.88	-0.69	0.58	1.74	0.058191	-0.71	0.59	2.07	0.002005	6.825-6.922
	7.16	-0.76	0.67	1.57	NS	-0.77	0.65	2.13	0.002562	7.133-7.194
Phenylalanine	7.31	-0.74	0.76	1.41	NS	-0.74	0.74	1.94	0.000639	7.272-7.331
	7.41	-0.71	0.62	1.42	NS	-0.70	0.58	2.14	0.003530	7.3727-7.441
Hipoxantine	8.17	-0.69	0.58	1.59	0.018693	-0.73	0.61	3.11	0.000876	8.144-8.180
	8.19	-0.65	0.53	1.40	0.070550	-0.59	0.49	1.69	0.011879	8.180-8.204

* the spectral integrals obtained using automatic signal deconvolution technique

Table S2. Compounds contributing to discrimination between the samples representative of cancer and normal ovary tissue (OPLS-DA $1_{j\text{-}Res}$ model) and between the samples representative of cancer and benign tumors (OPLS-DA $2_{J\text{-}res}$ model). The fold changes were computed as ratios of the median metabolite levels in the cancerous tissue to the median metabolite levels in the normal ovary or in the benign tumors, the p-values were obtained from the multiple comparisons of the mean ranks following the Kruskal-Wallis test.

NS – not significant, ND – not determined, p(corr)[1] - loadings scaled as correlation coefficients between the original data and scores obtained for the first component, VIP - Variable Importance at Projection

**Metabolite**	**Chemical shift [ppm]**	**HGSOC vs normal ovary**				**HGSOC vs benign tumors (fibrotic stroma)**				**Integral region [ppm]**
		**Model OPLS-DA $1_{j\text{-}Res}$**		**Univariate analysis**		**Model OPLS-DA $1_{j\text{-}Res}$**		**Univariate analysis**		
		**p(corr)[1]**	**VIP**	**Fold change**	**p value**	**p(corr)[1]**	**VIP**	**Fold change**	**p value**	
Isoleucine	0.93	-0.85	1.27	3.70	0.001213	-0.82	1.27	4.58	0.000249	0.917-0.935
	1.00	-0.87	1.26	3.29	0.000592	-0.83	1.24	4.34	0.000124	0.989-1.011
Leucine	0.94	-0.81	0.93	4.59	0.000906	-0.81	0.95	4.74	0.000380	0.935-0.950
	0.96	-0.85	0.99	4.32	0.000596	-0.81	0.93	4.50	0.000972	0.950-0.963
Valine	0.97	-0.80	1.92	2.21	0.000853	-0.76	1.86	2.04	0.000596	0.963-0.987
	1.03	-0.80	1.83	2.08	0.000403	-0.77	1.81	2.10	0.000409	1.017-1.050
Lactate	1.32	-0.77	11.86	1.95	0.010631	-0.75	11.87	2.40	0.004757	1.303-1.346
	4.11	-0.77	4.78	2.26	0.005947	-0.76	4.79	3.07	0.002689	4.09-4.15
Alanine	1.47	-0.91	5.74	4.14	0.000032	-0.89	5.60	4.27	0.000075	1.452-1.480
Acetate	1.91	-0.5	1.15	3.36	NS	-0.5	1.48	2.21	NS	1.897-1.923
N-acetylasparate	2.01	-0.48	1.89	33.38	0.000095	-0.50	2.01	18.91	0.017451	1.998-2.018
Nucleotide UDP sugars	2.07	-0.82	1.35	34.86	0.000187	-0.80	1.30	30.73	0.007409	2.063-2.076

Glutamine/Glutamate/Methionine	2.12	-0.73	1.75	6.26	0.000288	-0.71	1.77	8.04	0.000137	2.11-2.14
	2.13	-0.60	1.24			-0.56	1.18			
Glutamate	2.34	-0.76	1.72	2.20	0.000351	-0.65	1.49	2.23	0.003108	2.320-2.354
Succinate	2.41	-0.61	1.64	2.16	NS	-0.45	1.55	1.45	NS	2.390-2.409
Glutamine	2.44	-0.57	0.71	8.91	0.000317	-0.58	0.73	9.62	0.000240	2.430-2.450
Hipotaurine	2.64	-0.61	1.10	129.15	0.001634	-0.56	1.03	15.34	0.035340	2.631-2.656
Creatine	3.02	-0.63	3.96	2.02	0.008315	-0.60	3.93	3.02	0.000323	3.003-3.037
Ethanolamine	3.12	-0.60	1.05	6.50	0.005206	-0.36	0.61	3.16	NS	3.114-3.13
	3.81	-0.64	0.78	ND	ND	-0.29	0.35	ND	ND	-
Phosphocholine	3.21	-0.48	3.82	2.43	NS	-0.62	4.92	1.99	NS	3.205-3.218
Taurine	3.41	0.52	3.69	0.43	NS	0.52	3.44	0.37	NS	3.392-3.434
Glycine	3.55	-0.91	8.36	3.95	0.000104	-0.85	7.83	3.77	0.000227	3.533-3.562
Phosphoethanolamine/ Serine	3.99	-0.60	1.28	1.59	NS	-0.58	1.28	1.73	NS	3.994-3.998
Threonine	4.25	-0.86	0.87	12.23	0.000025	-0.83	0.84	6.35	0.001437	4.236-4.258
Ascorbate	4.51	0.48	1.12	0.53	NS	0.61	1.66	0.31	NS	4.491-4.532

Table S3. Compounds contributing to discrimination between the samples representative of cancer and fibrotic stroma in the malignant tumors (OPLS-DA 3_{CPMG} model). The fold changes were computed as ratios of the median metabolite level in the cancerous tissue to the median metabolite levels in the fibrotic stroma, the p-values were obtained from the multiple comparisons of the mean ranks following the Kruskal-Wallis test.

p(corr)[1] - loadings scaled as correlation coefficients between the original data and scores obtained for the first component, VIP - Variable Importance at Projection

<table>
<tr><th rowspan="2">Metabolite</th><th rowspan="2">Chemical shift [ppm]</th><th colspan="2">OPLS-DA 3_{CPMG}</th><th colspan="2">Univariate analysis</th><th rowspan="2">Integral region [ppm]</th></tr>
<tr><th>P(corr)[1]</th><th>VIP</th><th>Fold change</th><th>P value</th></tr>
<tr><td>Ethanolamine</td><td>3.12</td><td>0.60</td><td>1.08</td><td>1.81</td><td>0.065436</td><td>3.098-3.151</td></tr>
<tr><td rowspan="2">Phosphocholine / Glycerophosphocholine</td><td>3.21</td><td>0.80</td><td>6.93</td><td rowspan="2">3.33</td><td rowspan="2">0.008734</td><td rowspan="2">3.205-3.228</td></tr>
<tr><td>3.22</td><td>0.73</td><td>8.46</td></tr>
<tr><td>Glycine</td><td>3.55</td><td>0.74</td><td>5.13</td><td>1.80</td><td>0.084740</td><td>3.544-3.560</td></tr>
<tr><td rowspan="4">Phosphoethanolamine/Serine</td><td>3.96</td><td>0.74</td><td>2.22</td><td rowspan="4">1.79</td><td rowspan="4">0.029700</td><td rowspan="4">3.934-4.005</td></tr>
<tr><td>3.97</td><td>0.86</td><td>2.52</td></tr>
<tr><td>3.98</td><td>0.94</td><td>3.13</td></tr>
<tr><td>3.99</td><td>0.96</td><td>3.10</td></tr>
<tr><td>Lactate</td><td>4.12</td><td>0.86</td><td>4.90</td><td>1.68</td><td>0.097054</td><td>4.074-4.160</td></tr>
<tr><td>Threonine</td><td>4.25</td><td>0.93</td><td>1.23</td><td>1.34</td><td>0.050144</td><td>4.20-4.280</td></tr>
<tr><td>UDP/UTP/UMP/ Nucleotide UDP sugars</td><td>5.97</td><td>0.79</td><td>0.80</td><td>5.33</td><td>0.015830</td><td>5.924-6.013</td></tr>
<tr><td>UDP/ UTP/ Nucleotide UDP sugars</td><td>7.95</td><td>0.77</td><td>0.61</td><td>3.02</td><td>0.019963</td><td>7.916-8.002</td></tr>
</table>

Table S4. Compounds contributing to discrimination between the samples representative of cancer and fibrotic stroma in the malignant tumors (OPLS-DA $3_{J\text{-res}}$ model). The fold changes were computed as ratios of the median metabolite level in the cancerous tissue to the median metabolite level in the fibrotic stroma, the p-values were obtained from the multiple comparisons of the mean ranks following the Kruskal-Wallis test.

p(corr)[1] - loadings scaled as correlation coefficients between the original data and scores obtained for the first component, VIP - Variable Importance at Projection

<table>
<tr><th rowspan="2">Metabolite</th><th rowspan="2">Chemical shift [ppm]</th><th colspan="2">OPLS-DA $3_{J\text{-res}}$</th><th colspan="2">Univariate analysis</th><th rowspan="2">Integral region [ppm]</th></tr>
<tr><th>P(corr)[1]</th><th>VIP</th><th>Fold change</th><th>P value</th></tr>
<tr><td>Valine</td><td>1.03</td><td>0.69</td><td>1.47</td><td>1.60</td><td>0.073121</td><td>1.017-1.050</td></tr>
<tr><td rowspan="2">Lactate</td><td>1.32</td><td>0.91</td><td>13.24</td><td>2.14</td><td>0.051669</td><td>1.303-1.346</td></tr>
<tr><td>4.11</td><td>0.92</td><td>5.24</td><td>2.13</td><td>0.055940</td><td>4.09-4.15</td></tr>
<tr><td>Nucleotide UDP sugars</td><td>2.07</td><td>0.63</td><td>0.94</td><td>16.81</td><td>0.022622</td><td>2.063-2.076</td></tr>
<tr><td>Glutamine</td><td>2.44</td><td>0.64</td><td>0.56</td><td>3.96</td><td>0.033504</td><td>2.430-2.450</td></tr>
<tr><td>Creatine</td><td>3.02</td><td>0.55</td><td>3.29</td><td>1.96</td><td>0.089658</td><td>3.003-3.037</td></tr>
<tr><td rowspan="2">Phosphocholine/
Glycerophosphocholine</td><td>3.21</td><td>0.69</td><td>5.67</td><td rowspan="2">4.76</td><td rowspan="2">0.002033</td><td rowspan="2">3.205-3.218</td></tr>
<tr><td>3.22</td><td>0.53</td><td>6.40</td></tr>
<tr><td rowspan="3">Phosphoethanolamine/Serine</td><td>3.96</td><td>0.59</td><td>1.28</td><td rowspan="3">1.97</td><td rowspan="3">0.007019</td><td rowspan="3">3.994-3.998</td></tr>
<tr><td>3.97</td><td>0.54</td><td>0.94</td></tr>
<tr><td>3.99</td><td>0.67</td><td>1.36</td></tr>
<tr><td>Threonine</td><td>4.25</td><td>0.78</td><td>0.70</td><td>2.75</td><td>0.064176</td><td>4.236-4.258</td></tr>
</table>

Table S5. Compounds contributing to discrimination between the samples representative of fibrotic stroma in the malignant tumors and fibrotic stroma in the benign tumors (OPLS-DA 4_{CPMG} model). The fold changes were computed as ratios of the median metabolite levels in the fibrotic stroma in the malignant tumors to those levels in the fibrotic stroma in the benign tumors. the p-values were obtained from the multiple comparisons of the mean ranks following the Kruskal-Wallis test.

p(corr)[1] - loadings scaled as correlation coefficients between the original data and scores obtained for the first component, VIP - Variable Importance at Projection

Metabolite	Chemical shift [ppm]	OPLS-DA 4_{CPMG}		Univariate analysis		Integral region [ppm]
		p(corr)[1]	VIP	Fold change	P value	
Alanine	1.47	-0.67	4.27	2.09	0.008727	1.422-1.514
Lysine	1.73	-0.65	1.36	1.38	0.032324	1.642-1.776
Acetate	1.91	-0.57	2.06	1.38	0.014476	1.90-1.923
Glutamate	2.34	-0.55	2.58	1.92	0.040963	2.303-2.374
Glutamine	2.44	-0.60	1.60	1.55	0.021377	2.407-2.472
2.60–2.70 ppm	2.62	-0.67	1.19	ND	ND	-
	2.64	-0.46	0.99	ND	ND	
	2.68	-0.59	0.94	ND	ND	
	2.70	-0.60	0.76	ND	ND	
Choline	3.20	0.63	9.61	0.53	NS	3.182-3.202
Taurine	3.41	0.71	7.29	0.55	NS	3.376-3.442
Glycine	3.55	-0.48	4.68	1.70	NS	3.544-3.560
Ascorbate	4.51	0.60	2.57	0.61	NS	4.494-4.529
Inosine	6.08	0.54	0.54	ND	ND	-
	8.22	0.35	0.46	ND	ND	-
Uracil	5.79	-0.33	0.24	1.57	NS	5.748-5.810
	7.53	-0.54	0.35	3.47	NS	7.470-7.566
Tyrosine	6.88	-0.65	0.61	1.53	0.099946	6.825-6.922
	7.18	-0.59	0.58	1.82	NS	7.133-7.194
Phenylalanine	7.31	-0.62	0.72	1.55	0.056669	7.272-7.331
	7.41	-0.64	0.64	1.75	NS	7.3727-7.441

Table S6. Compounds contributing to discrimination between the samples representative of fibrotic stroma in the malignant tumors and fibrotic stroma in the benign tumors (OPLS-DA 4j-Res model). The fold changes were computed as ratios of the median metabolite levels in the fibrotic stroma in the malignant tumors to those levels in the fibrotic stroma in the benign tumors, the p-values were obtained from the multiple comparisons of the mean ranks following the Kruskal-Wallis test.

NS – not significant, p(corr)[1] - loadings scaled as correlation coefficients between the original data and scores obtained for the first component, VIP - Variable Importance at Projection

**Metabolite**	**Chemical shift [ppm]**	**OPLS-DA $4_{J\text{-res}}$**		**Univariate analysis**		**Integral region [ppm]**
		**p(corr)[1]**	**VIP**	**Fold change**	**P value**	
Isoleucine	0.93	-0.74	1.87	2.74	0.034366	0.917-0.935
	1.00	-0.70	1.27	2.27	0.108977	0.989-1.011
Leucine	0.94	-0.65	1.21	2.52	0.081991	0.935-0.950
	0.96	-0.66	1.23	2.44	0.049252	0.950-0.963
Valine	0.97	-0.50	1.76	1.41	NS	0.963-0.987
	1.03	-0.53	1.79	1.31	NS	1.017-1.050
Alanine	1.47	-0.63	4.65	1.76	NS	1.452-1.480
Acetate	1.91	-0.61	2.31	2.07	NS	1.897-1.923
Glutamate	2.34	-0.49	1.66	1.67	NS	2.320-2.354
Glutamine	2.44	-0.53	0.92	2.43	NS	2.430-2.450
Taurine	3.41	0.63	7.15	0.30	NS	3.392-3.434
Glycine	3.55	-0.61	7.52	1.65	NS	3.533-3.562
Threonine	4.25	-0.62	0.68	2.31	NS	4.236-4.258
Ascorbate	4.51	0.59	2.87	0.39	NS	4.491-4.532

Table S7. The p(corr)[1] (loadings scaled as correlation coefficients between the original data and scores obtained for the first component) and VIP (Variable Importance at Projection) values for the most important metabolites in OPLSR $_{CPMG}$ model.

**Metabolite**	**Chemical shift [ppm]**	**p(corr)[1]**	**VIP**
Alanine	1.46	0.53	3.83
Glutamine	2.44	0.50	1.18
Hipotaurine/Methionine	2.62	0.64	1.06
	2.64	0.67	1.36
Creatine	3.02	0.60	3.31
	3.92	0.69	2.70
Ethanolamine	3.12	0.55	1.42
Choline	3.20	0.54	4.73
Phosphocholine	3.21	0.92	10.08
Glycerophosphocholine	3.22	0.87	8.58
Mio-inositol	3.51	0.58	2.94
	3.53	0.56	3.75
	3.61	0.62	4.79
	4.05	0.58	3.18
Glycine	3.55	0.61	6.06
Phosphoethanolamine/serine	3.96	0.52	1.95
	3.97	0.74	2.83
	3.98	0.91	3.65
	3.99	0.77	3.06
Lactate	4.12	0.72	5.48
Threonine	4.25	0.55	0.98
Glutathione	4.57	0.58	0.89
UDP/UTP/ UMP/Nucleotide UDP sugars	5.97	0.67	0.75
UDP/ UTP/Nucleotide UDP sugars	7.95	0.68	0.58
UMP	8.08	0.51	0.46
N?	6.11	0.68	0.39

Table S8. The p(corr)[1] (loadings scaled as correlation coefficients between the original data and scores obtained for the first component) and VIP (Variable Importance at Projection) values for the most important metabolites in OPLSR $_{J\text{-res}}$ model.

Metabolite	Chemical shift [ppm]	p(corr)[1]	VIP
Lactate	1.32	0.52	10.37
	4.11	0.57	4.49
N-acetylasparate	2.01	0.55	2.70
	2.70	0.56	0.72
Nucleotide UDP sugars	2.07	0.67	1.28
Methionine/glutamine	2.12	0.66	1.72
Glutamine	2.43	0.84	1.04
Hipoturine	2.64	0.61	1.34
Ethanolamine	3.12	0.57	0.79
Phosphocholine	3.21	0.78	8.10
Glycerophosphocholine	3.22	0.84	7.46
Myo-inositol	3.53	0.52	1.46
	3.61	0.63	3.49
	4.05	0.62	2.82
Phosphoethanolamine/serine	3.97	0.43	0.98
	3.99	0.55	1.28

Table S9. Results of linear regression analysis between the metabolite levels determined using HR MAS p-Jres NMR spectra and cancer cell fraction.

**Metabolite (Chemical shift)**	**Pearson correlation coefficient r, p value**	**Integral region [ppm]**
Lactate (4.11 ppm)	r = 0.52; p = 0.0072	1.303-1.346
Lactate (1.32 ppm)	r = 0.49; p = 0.0125	4.09-4.15
Myo-inositol (4.05 ppm)	r = 0.48; p = 0.0155	4.035-4.062
Phosphoethanolamine/Serine (3.97 ppm)	r = 0.68; p = 0.0002	3.944-3.998
Glycine (3.54 ppm)	r = 0.42; p = 0.0346	3.533-3.562
Phosphocholine (3.21 ppm)/ Glycerophosphocholine (3.22 ppm)	r = 0.85; p = 0.00000	3.205-3.218
Creatine (3.02 ppm)	r = 0.41; p = 0.0415	3.003-3.037
Ethanolamine (3.12)	r = 0.40; p = 0.0464	3.114-3.13
Hipotaurine (2.64 ppm)	r = 0.47; p = 0.0172	2.631-2.656
Glutamine (2.44 ppm)	r = 0.71; p = 0.00006	2.430-2.450
Succinate (2.41 ppm)	r = 0.63; p = 0.0008	2.390-2.409
Nucleotide UDP sugars (2.07 ppm)	r = 0.61; p = 0.0012	2.063-2.076
N-acetylasparate (2.01 ppm)	r = 0.47; p = 0.0184	1.998-2.018
Alanine (1.47 ppm)	r = 0.41; p = 0.0412	1.452-1.480
